# Synthetic control and empirical prediction of redox potentials for Co_4_O_4_ cubanes over a 1.4 V range: implications for catalyst design and evaluation of high-valent intermediates in water oxidation†
†Electronic supplementary information (ESI) available. CCDC 1532026–1532032. For ESI and crystallographic data in CIF or other electronic format see DOI: 10.1039/c7sc00627f



**DOI:** 10.1039/c7sc00627f

**Published:** 2017-04-07

**Authors:** Andy I. Nguyen, Jianing Wang, Daniel S. Levine, Micah S. Ziegler, T. Don Tilley

**Affiliations:** a Department of Chemistry , University of California , Berkeley , California 94720-1460 , USA . Email: tdtilley@berkeley.edu; b Chemical Sciences Division , Lawrence Berkeley National Laboratory , Berkeley , California 94720 , USA

## Abstract

The oxo-cobalt cubane unit [Co_4_O_4_] is of interest as a homogeneous oxygen-evolution reaction (OER) catalyst, and as a functional mimic of heterogeneous cobalt oxide OER catalysts. The synthesis of several new cubanes allows evaluation of redox potentials for the [Co_4_O_4_] cluster, which are highly sensitive to the ligand environment and span a remarkable range of 1.42 V. The [CoIII4O_4_]^4+^/[CoIII3Co^IV^O_4_]^5+^ and [CoIII3Co^IV^O_4_]^5+^/[CoIII2CoIV2O_4_]^6+^ redox potentials are reliably predicted by the p*K*_a_s of the ligands. Hydrogen bonding is also shown to significantly raise the redox potentials, by ∼500 mV. The potential-p*K*_a_ correlation is used to evaluate the feasibility of various proposed OER catalytic intermediates, including high-valent Co-oxo species. The synthetic methods and structure–reactivity relationships developed by these studies should better guide the design of new cubane-based OER catalysts.

## Abstract 2

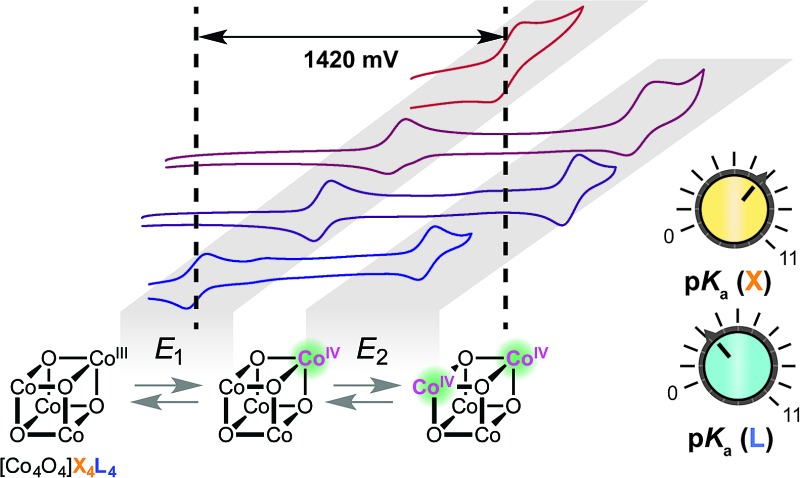
The oxo-cobalt cubane unit [Co_4_O_4_] is of interest as a homogeneous oxygen-evolution reaction (OER) catalyst, and as a functional mimic of heterogeneous cobalt oxide OER catalysts.

## Introduction

Research on catalysts for the oxygen evolution reaction (OER), motivated by the goal of creating an artificial photosynthesis system, has generated a number of hypotheses that challenge the traditional limits of transition-metal oxidation states and bonding. Thus, catalytic OER cycles often invoke unusually high and rare oxidation states for the metal centers, which are bound to reactive terminal oxo ligands.^1–9^ In particular, for intensely studied cobalt-based OER catalysts, a Co^IV^-oxo intermediate is commonly invoked.^3,6,9^ However, the Co^IV^ oxidation state is quite rare, and ligand field considerations seem to suggest a high instability for a terminal oxo ligand bound to Co^IV^.^10^ Recently, several experiments have confirmed the presence of Co^IV^ species during water oxidation catalysis, but evidence for a discreet, terminal Co^IV^ oxo species remains indirect.^3,9,11,12^ Remarkably, many density functional theory (DFT) calculations of cobalt-catalyzed OER suggest an even more unconventional species, a Co^V^-oxo intermediate that is sometimes described as a Co^IV^-oxyl radical.^4,5^ Experimental evidence for this type of intermediate is provided by kinetic analysis of OER mediated by a molecular Co_4_O_4_ cubane, which is a rare example of a structural and functional model for the active site of OER catalysis.^6*a*^ For the oxo cubane Co_4_(μ-O)_4_(OAc)_4_(py)_4_ a singly oxidized state, formally CoIII3Co^IV^, is firmly established by isolation and spectroscopy.^3,6a^ The doubly oxidized state, formally CoIII2CoIV2, is observed at highly positive potentials, by cyclic voltammetry.^13^ While the kinetic analysis points to involvement of the doubly oxidized state in the oxygen evolution mechanism, the nature of intermediates associated with this oxidation state remains largely unknown.

For evaluation of possible CoIII2CoIV2 (or alternatively CoIII3Co^V^) intermediates, it would be quite useful to establish reliable methods for predicting the redox potentials associated with particular coordination environments. For example, the Co_4_O_4_ cubane Co_4_(μ-O)_4_(OAc)_4_(py)_4_ has been shown to form the hydroxide complex [Co_4_O_4_(OAc)_3_(OH)_2_(py)_4_]^–^ by displacement of acetate, and kinetic studies on OER indicate that this species is oxidized to high-valent species.^6^ The resulting, transient intermediates have not been directly observed; therefore, alternative, indirect experimental methods for evaluation of candidate structures are useful. As shown here, a strategy for estimation of Co^IV^/Co^V^ redox potentials for transitory intermediates is based on extrapolation of linear free-energy relationships (LFERs). This analysis requires a large and diverse set of related cubanes with various ligand sets and redox potentials, to provide a useful LFER from which redox properties can be confidently predicted.

In this report, we demonstrate that the Co_4_O_4_ core is readily and precisely manipulated to tune its chemical and electronic properties over an unprecedented range. While the parent cluster Co_4_O_4_(OAc)_4_(py)_4_ has been well-studied, its controlled structural modification has never before been demonstrated; such clusters are generally prepared by “self-assembly” methods rather than by rational syntheses.^14^ Synthetic methods were used to obtain electron-rich or electron poor cubanes, cubanes with mixed-carboxylate ligand sets, and cubanes possessing secondary-sphere hydrogen-bond donors. It is noteworthy that this synthetic methodology allows introduction of secondary-sphere hydrogen bonding into the cubane structure since the role of hydrogen bonding in electron transfer and water oxidation (especially in the OEC of photosystem II) has been an important topic for many years.^1b–h^

Analysis of a substantial library of oxo cubanes provides empirical linear correlations between ligand p*K*_a_ values and redox potentials for singly and doubly oxidized species, over a range of 1.42 V. This analysis also quantifies the effect of hydrogen bonding on redox properties in this cubane system. These relationships offer a useful predictive tool for evaluating potential intermediates in water-splitting mechanisms. They should also provide important guidance in catalyst design studies for OER.

## Results and discussion

### Synthesis of new cobalt oxo cubanes

The synthesis of Co_4_O_4_ cubanes has previously been accomplished by the “self-assembly” route.^14*b*^ While this method is simple to execute, its harshly oxidizing conditions and less predictable nature prevents the targeted synthesis of many structurally diverse cubane clusters. Notably, the “self-assembly” route only leads to symmetrically ligated cubanes, and has only been demonstrated with ligands that are not very electron rich or electron poor. Given potentially important applications of the Co_4_O_4_ cubane unit (*vide supra*), general synthetic routes that allow ready access to a diverse library of cubanes, including unsymmetrically substituted, electron poor and electron rich examples, are desired.

The various cubane complexes to be discussed in this study possess a Co_4_O_4_ cubane core with different ligand sets that are described in a concise manner by a convenient descriptor. Compounds of the general formula [CoIII4O_4_X_*x*_L_*y*_]^(4–*x*)+^ are abbreviated **[*x*X–*y*L]^(4–^*^x^*^)+^**, where *x* and *y* are integers equal or greater than 0, and denote the stoichiometry of **X** (anionic ligand) and **L** (neutral ligand), respectively. If multiple types of **X** or **L** are present in the same complex, then the additional ***x*X** or ***y*L** is appended in the fashion **[*x*_1_X_1_,*x*_2_X_2_,…*x_n_*X*_n_*–*y*_1_L_1_,*y*_2_L_2_,…*y_n_*L*_n_*]^(4–^*^x^*^)+^**. If the [Co_4_O_4_]^4+^ unit is oxidized to [Co_4_O_4_]^5+^ or [Co_4_O_4_]^6+^, the notation changes to **[*x*X–*y*L]^(5–^*^x^*^)+^** and **[*x*X–*y*L]^(6–^*^x^*^)+^**, respectively. For example, a cubane with the formula [Co_4_O_4_(OAc)_2_(OH)(H_2_O)_3_(py)_4_]^+^ is abbreviated **[2OAc,OH-3H_2_O,4py]^+^**, and its successive oxidation products are **[2OAc,OH-3H_2_O,4py]^2+^** and **[2OAc,OH-3H_2_O,4py]^3+^**.

**Chart 1:**
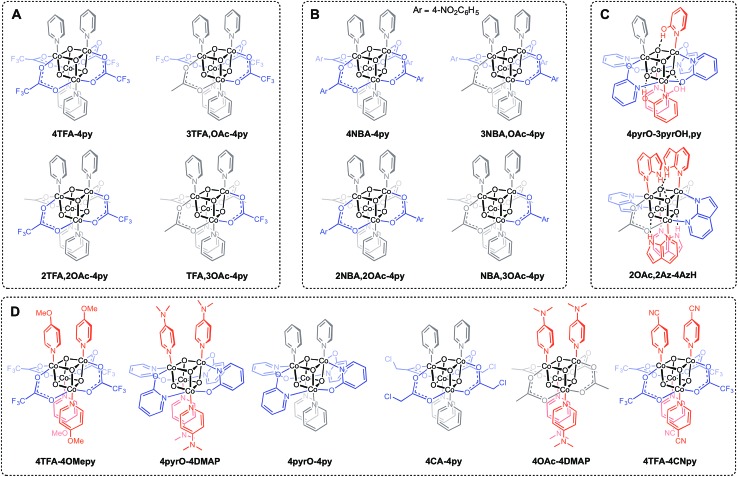
New cubane complexes. (A) Trifluoroacetate (TFA) series synthesized by method C; (B) *p*-nitrobenzoate (NBA) series synthesized by method C; (C) hydrogen-bonded cubanes; (D) cubanes synthesized by a methods B and/or C.

Three general methods were used to synthesize the new cubane complexes, shown in Chart 1. The “self-assembly” route (method A, eqn (1)), patterned after the Das synthesis of Co_4_O_4_(OAc)_4_(py)_4_ from Co(NO_3_)_2_(H_2_O)_6_, NaX, L, and H_2_O_2_, has been used to synthesize a small number of closely related cubanes such as Co_4_O_4_(OAc)_4_(*p*-cyanopyridine)_4_ (**4-OAc-4CNpy**).^14b,20^ However, attempts to extend this method to many of the cubane derivatives described below were unsuccessful. Thus, this does not appear to be a generally successful synthetic strategy for the synthesis of new cubane complexes.

**Fig. 1:**
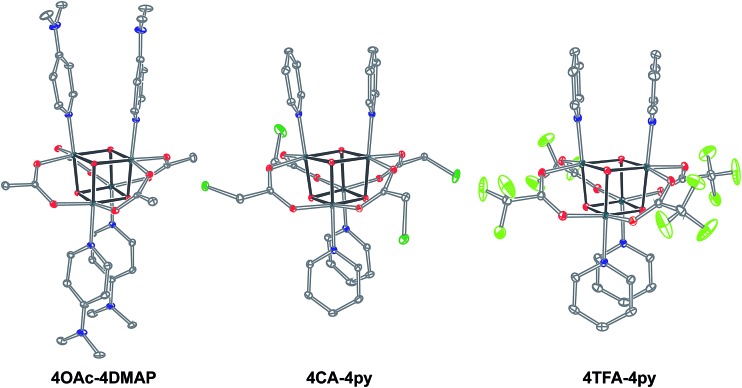
Single-crystal XRD structures of cubane complexes. Thermal ellipsoids are at 30% probability, with hydrogen atoms and solvent molecules omitted for clarity.

Exchange of neutral ligands (method B, eqn (2)) is useful in the synthesis of cubanes with L = DMAP. The cubane Co_4_O_4_(OAc)_4_(DMAP)_4_ (**4-OAc-4DMAP**) was produced in this way, in 67% recrystallized yield. Method B works well in other cases (*vide infra*), under conditions where p*K*_a_(L′H^+^) > p*K*_a_(LH^+^). Similarly, synthesis of the cubane with X′ = CF_3_COO^–^ was achieved by heating **4OAc-4py** with a slight excess of CF_3_CO_2_H to give Co_4_O_4_(O_2_CCF_3_)_4_(py)_4_ (**4TFA-4py**) in 56% yield. This procedure (method C, eqn (3)) is driven by release of HX; thus, it works well when p*K*_a_(HX') < p*K*_a_(HX). Methods B and C provide predictable and divergent routes for cubane diversification (Chart 1), and in contrast to method A, give crude products that are mostly free of Co(ii) impurities, as determined by thin-layer chromatography. Remarkably, substitution of ligands by methods B and C is highly stereoselective, always placing the X ligands around equatorial faces and the L ligands on the “top” and “bottom” faces, as evidenced by NMR spectroscopy and crystallography (Fig. 1).1
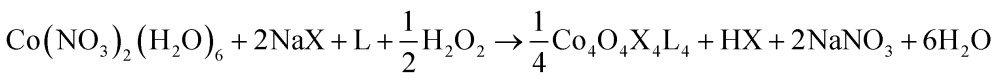

2
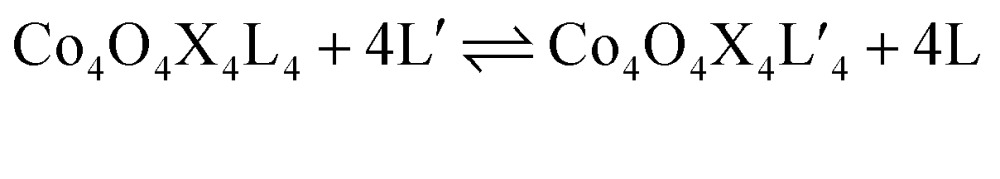

3
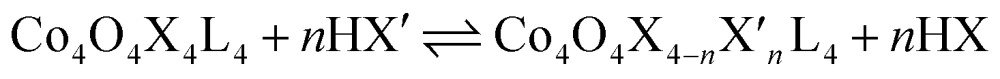



**Fig. 2:**
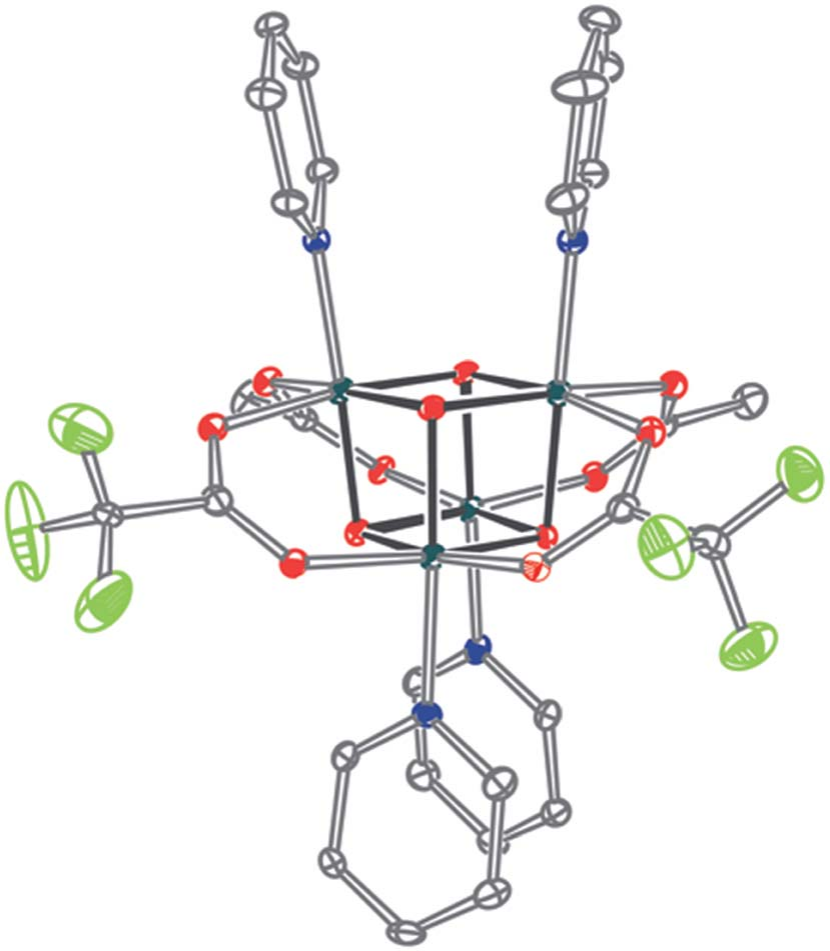
Single-crystal X-ray structure of **2TFA,2OAc-4py**. Thermal ellipsoids shown at 30% probability. Hydrogen atoms were omitted for clarity.

Significantly, method C allows for the synthesis of mixed-carboxylate cubanes. Mono-, di-, or tri-substituted carboxylate cubanes were produced using two RCO_2_H equivalents per cubane. This mixture of mono-, di-, tri-, and tetra-substituted cubanes is generally separable by column chromatography since the compounds are highly colored and exhibit significantly different *R*_f_ values. By this method, Co_4_O_4_(OAc)_4–*n*_(CF_3_CO_2_)_*n*_(py)_4_ (**nTFA,(4-n)OAc-4py**) and Co_4_O_4_(OAc)_4–*n*_(*p*-nitrobenzoate)_*n*_(py)_4_ (**nNBA,(4-n)OAc-4py**) complexes, where *n* = 1, 2, and 3, have been synthesized and fully characterized. Interestingly, the formation of disubstituted cubanes is stereoselective for *cis*-substitution – that is the two carboxylate (X′) ligands are on adjacent faces of the cubane, as revealed by X-ray crystallography of **2TFA,2OAc-4py** (Fig. 2). The ^1^H NMR spectra reveal the presence of only one product, suggesting that the crystal structures represent the bulk material.

**Scheme 1:**
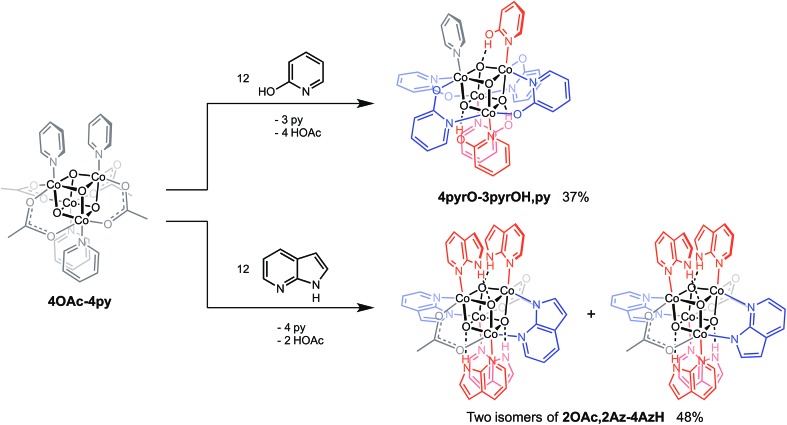
Synthesis of cubane complexes containing intramolecular hydrogen-bonding.

**Fig. 3:**
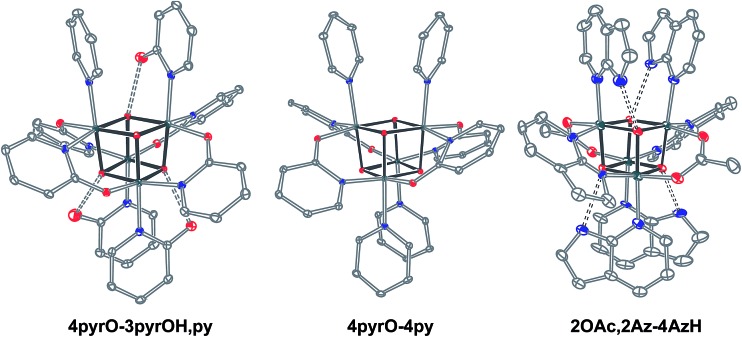
Single-crystal XRD structures of cubanes containing pyrindonate or 7-azaindolate ligands. Intramolecular hydrogen-bond donor–acceptor distances are distinguished as dashed-bonds. Thermal ellipsoids shown at 30% probability. Hydrogen atoms and solvent are omitted for clarity.

Cubanes bearing electron-rich X ligands 2-pyridonate (p*K*_a_(O–H) = 8.05)^15^ and 7-azainodolate (p*K*_a_(N^1^–H) ∼ 15)^16^ were synthesized in a manner similar to that of method C, but the products have a somewhat different composition (Scheme 1). Thus, heating **4OAc-4py** with excess 2-hydroxypyridine in acetonitrile gave Co_4_O_4_(2-pyridonate)_4_(2-hydroxypyridine)_3_(py) (**4pyrO-3pyrOH,py**) in 37% yield, after column chromatography. Elemental analysis and mass spectrometry are consistent with the stoichiometries determined by NMR spectroscopy. Similarly, heating **4OAc-4py** with an excess of 7-azaindole in acetonitrile gave Co_4_O_4_(OAc)_2_(7-azaindolate)_2_(7-azaindole)_4_ (**2OAc,2Az-4AzH**) in 48% yield. Though the p*K*_a_ values of 2-hydroxypyridine and 7-azaindole are higher than those of HOAc, the reaction is driven by use of excess ligand and the insolubilities of **4pyrO-3pyrOH,py** and **2OAc,2Az-4AzH** in acetonitrile. These reactions provide different types of cubane structures, in that protonated versions of the X ligands serve as the L ligands (2-hydroxypyridine and 7-azaindole possess pyridyl nitrogen atoms), and substantial substitution of the L-positions on the cubane is observed. Interestingly, the protonated, L-type ligands engage in hydrogen bonding with their O–H or N–H bonds interacting with the μ_3_-oxo ions of the [Co_4_O_4_] core. The 2-hydroxypyridine ligand can be substituted for other L ligands by method B to produce **4pyrO-4py** and **4pyrO-4DMAP**. The latter two compounds were characterized by ^1^H NMR spectroscopy and single-crystal X-ray diffraction (Fig. 3). ^1^H NMR spectroscopy suggests that **4pyrO-3pyrOH,py** coexists as C_1_-symmetric species in solution at room temperature, suggesting that hydrogen-bonds slow the rotation about the Co–N bonds. For **2OAc,2Az-4AzH**, two C_2_-symmetric isomers co-crystallized, and differ by the relative orientation of the 7-azin ligands. The O(X)···O(X) distances of 2.44 Å in **4pyrO-3pyrOH,py**, and the N(X)···O(X) distances of 2.69 Å in **2OAc,2Az-4AzH** are consistent with strong to moderate hydrogen bonding.^17^ Thus, these cubanes possess an additional secondary coordination sphere that affects the structural properties of the cubane core. Secondary coordination sphere effects are highly important in biology, and mimicry of this feature in inorganic complexes is a long-standing challenge that often involves special ligand design.^1*c*^

### Electrochemistry

**Fig. 4:**
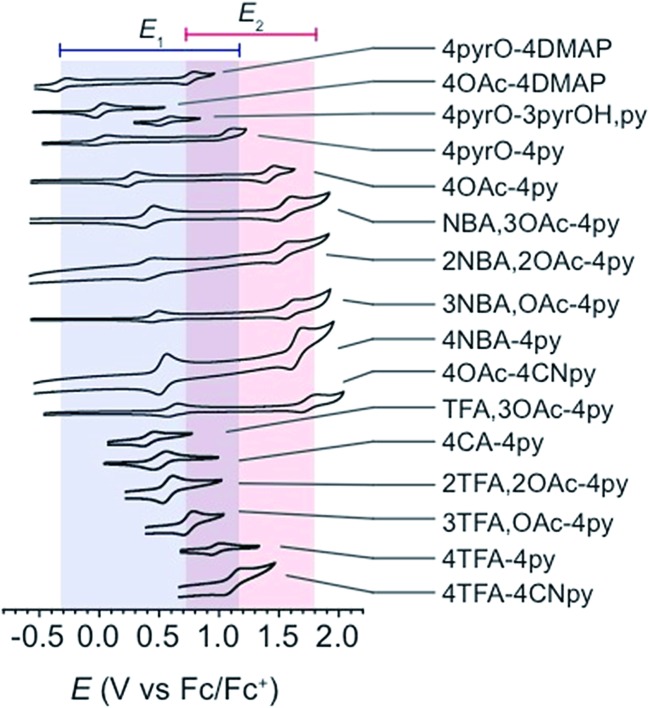
Cyclic voltammograms of cubane complexes, synthesized in this work, in organic solvents (see Table 1 for details, 100 mV s^–1^ scan rate, 0.1 M [^*n*^Bu_4_N]PF_6_ electrolyte). The blue and red boxes indicate the range of *E*_1_ and *E*_2_ potentials, respectively. For some cubanes, the *E*_2_ values lie beyond the solvent's electrochemical window.

All cyclic voltammograms of the cubanes exhibit a reversible event for the [CoIII4O_4_]^4+^/[Co^IV^CoIII3O_4_]^5+^ couple (*E*_1_), and depending on the ligand set, either a reversible or irreversible event for the [Co^IV^CoIII3O_4_]^5+^/{[Co^V^CoIII3O_4_]^6+^ ↔ [CoIV2CoIII2O_4_]^6+^} couple (*E*_2_, Fig. 4). The reversibility of *E*_2_ seems to depend on the combination of X and L, rather than the individual identities of X and L. In general, when there is a large asymmetry in the electron-donating properties of X and L (Δp*K*_a_ > 4), *E*_2_ is irreversible. Four examples that illustrate this trend are **4OAc-4DMAP**, **4pyrO-4py**, **4pyrO-4DMAP**, and **4OAc-4py**. The first two cubanes have two sets of ligands with Δp*K*_a_ > 4, and their cyclic voltammograms indicate an irreversible oxidation process at all measured scan rates (*E*_2_). However, the two sets of ligands in **4pyrO-4DMAP** and **4OAc-4py** have a Δp*K*_a_ < 2, and reversible *E*_2_ events. This observation may suggest that the *E*_2_ redox couple for cubanes with a ligand Δp*K*_a_ > 4 may involve some degree of ligand non-innocence. For example, if there is a large difference between the donating properties of X and L, electron–hole character may concentrate on the more donating ligand, leading to oxidative degradation. Conversely, when the X and L ligands are both good donors, the electron–hole character is distributed more evenly across all eight ligands to give a more stable complex. The electronic structure of the species resulting from this second oxidation will be discussed in later sections.

**Fig. 5:**
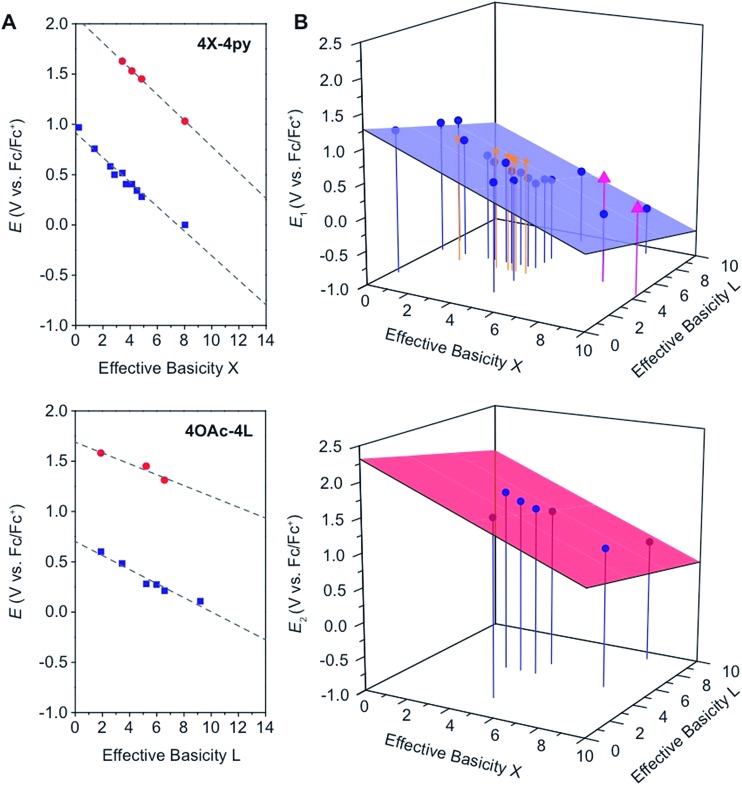
(A) Plots and fits of *E*_1_ and *E*_2_ potentials *versus* effective basicity for **4X-4py** (series 1, top) and **4OAc-4L** (series 2, bottom). The blue squares (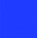) are the *E*_1_ values and the red circles (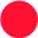) are the *E*_2_ values. The dashed lines are the linear fits. (B) Surface fits of *E*_1_ (eqn (5), top) and *E*_2_ (eqn (6), bottom) for all cubane complexes. The blue circles (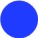) are redox potentials for **4X-4L** cubanes, the orange stars (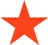) are *E*_1_ values for **[2X-4L]^2+^** from ref. 21, and the purple triangles (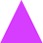) are potentials for the hydrogen-bonded cubanes. Vertical drop lines are presented as visual guides.

In general, and as expected, the observed redox couples reflect a dependence on the donor properties of the cubane ligand set, such that they shift anodically with electron-withdrawing ligands and cathodically with electron-donating ligands. To evaluate the influence of the L ligands on redox potentials, sixteen cubanes were analyzed holding X = OAc constant while varying L (**4OAc-4L**, series 1), and conversely holding L = py constant while varying X (**4X-4py**, series 2). The redox potentials for both series (1 and 2, respectively) correlate linearly with a convenient descriptor for the ligand donating ability, the average p*K*_a_ (aqueous)^15,16,18,19^ of HX and HL^+^ per cobalt center (eqn (4); Fig. 5A). This average p*K*_a_ value descriptor will herein be referred to as the effective basicity of the ligand set. Here, p*K*_a_ is employed as a free-energy representation for the electron-donating ability of the ligand. A previous report used Hammett parameters to correlate ligand donor ability with Co_4_O_4_ redox activity, but this analysis is limited to only substituted aryl-based ligands, whereas p*K*_a_ is a much more widely useful parameter.^20^

A plot of *E*_1_ *vs.* effective basicities for the complexes in series 1 is linear with a slope of –120 ± 7 mV dec^–1^, and series 2 also provides a linear relationship with a slope of –69 ± 9 mV dec^–1^. Similarly, the slopes of lines derived from the *E*_2_ values are very similar, at –128 ± 9 mV dec^–1^ (series 1) and –51 ± 9 mV dec^–1^ (series 2). The magnitudes of these slopes demonstrate that the redox couples for the cubanes are about twice as sensitive to the X *vs.* the L ligand. A possible cause for this effect will be discussed in the next section. Most surprising is that the redox potentials of unsymmetrical, mixed-carboxylate cubanes could be predicted simply by considering the summation of the p*K*_a_ values. This fact would seem to suggest that the Co_4_O_4_ cubane core levels out the electronic effects of coordination asymmetry through an electronically communicative mechanism. In this context, the four cobalt ions in the Co_4_O_4_ unit essentially behave as a single “superion” entity. Both series of potentials fit to planar surfaces (eqn (5) and (6), Fig. 5B) with *R* ^2^ values of 96% and 98%, respectively.4
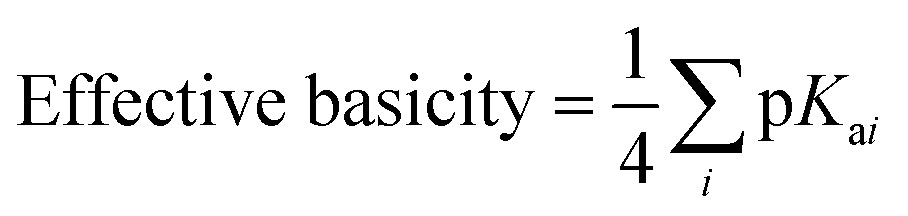

5
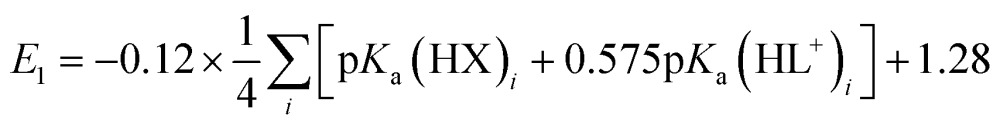

6
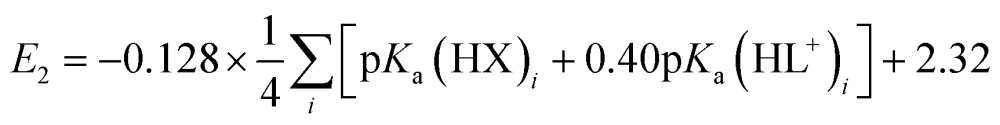



To explore the generality of eqn (5) and (6) derived from compound series 1 and 2, the *E*_1_ and *E*_2_ values for cubanes based on additional combinations of X and L were measured and compared to the predicted values. Including these additional combinations of X and L, the *E*_1_ redox potentials observed for tetracobalt oxo cubanes range from –0.30 to 1.12 V (a span of 1.42 V; Table 1). A smaller range of 830 mV was observed for *E*_2_, perhaps because some of the corresponding oxidation events exist outside the solvent's electrochemical window. Satisfyingly, the redox potentials of all the cubanes (without intramolecular hydrogen-bonds) are predicted by eqn (5) and (6) with maximum absolute errors of 0.099 and 0.050 V, respectively. In addition, reported redox potentials for cubanes of the type [Co_4_O_4_X_2_L24]^2+^ (L^2^ = 2,2′-bipyridine) follow the relationships described by eqn (5).^21^ This observation indicates that the relative number of X to L ligands does not affect the validity of eqn (5), and therefore the coefficients must reflect the intrinsic donor properties of X- *versus* L-type ligands. In summary, it appears that eqn (5) and (6) hold for (1) a wide range of X and L, (2) symmetric and asymmetric ligand coordination environments around the cubane, and (3) X ligands other than carboxylates.

**Table 1:** Cubane complexes and their experimental redox potentials *vs.* Fc/Fc^+^

Cubane	Effective basicity X^15,16,18,19^	Effective basicity L^15,16,18,19^	*E*_1_ (V)	*E*_2_ (V)	Solvent	Reference
**4TFA-4CNpy**	0.23	1.90	1.12		CH_2_Cl_2_	This work
**4TFA-4OMepy**	0.23	6.39	0.90		MeCN	This work
**4TFA-4py**	0.23	5.25	0.97		CH_2_Cl_2_	This work
**3TFA,OAc-4py**	1.37	5.25	0.76		CH_2_Cl_2_	This work
**2TFA,2OAc-4py**	2.54	5.25	0.58		CH_2_Cl_2_	This work
**TFA,3OAc-4py**	3.70	5.25	0.40		CH_2_Cl_2_	This work
**4CA-4py**	2.86	5.25	0.50		MeCN	This work
**4NBA-4py**	3.43	5.25	0.52	1.63	MeCN	This work
**3NBA,OAc-4py**	3.79	5.25	0.47	1.59	MeCN	This work
**2NBA,2OAc-4py**	4.15	5.25	0.41	1.53	MeCN	This work
**NBA,3OAc-4py**	4.50	5.25	0.34	1.48	MeCN	This work
**4OAc-4py**	4.86	5.25	0.28	1.45	MeCN	13
**4OAc-4CNpy**	4.86	1.90	0.60	1.58	MeCN	14
**4OAc-4COOEtpy**	4.86	3.45	0.48		MeCN	20
**4OAc-4OMe**	4.86	6.39	0.21	1.31^*b*^	MeCN	20
**4OAc-4Mepy**	4.86	5.99	0.27		MeCN	20
**4OAc-4DMAP**	4.86	9.20	0.11	1.10^*b*^	MeCN	This work
**4pyrO-3pyrOH,py**	8.04	5.23	0.55		CH_2_Cl_2_	This work
**4pyrO-4py**	8.04	5.25	0.0	1.03	CH_2_Cl_2_	This work
**4pyrO-4DMAP**	8.04	9.20	–0.30	0.80	CH_2_Cl_2_	This work
**2OAc,2Az-4AzH**	∼10	4.45	0.30^*b*^		DMF	This work
**[2OAc-4bipy]^2+^** ^*a*^	2.43	4.34	0.69		MeCN	21
**[2NBA-4bipy]^2+^** ^*a*^	1.71	4.34	0.77		MeCN	21
**[2BA-4bipy]^2+^** ^*a*^	2.10	4.34	0.70		MeCN	21
**[2PFBA-4bipy]^2+^**	0.80	4.34	0.86		MeCN	21
**[2CBA-4bipy]^2+^** ^*a*^	2.00	4.34	0.72		MeCN	21
**[2*t*BuBA-4bipy]^2+^** ^*a*^	2.19	4.34	0.68		MeCN	21

^*a*^bipy = 2,2-bipyridine, BA = benzoate, PFBA = pentafluorobenzoate, CBA = 4-chlorobenzoate, *t*BuBA = 4-^*t*^Butylbenzoate.

^*b*^Value obtained by differential pulse voltammetry (DPV).

### Effects of protic solvent and hydrogen bonding

The redox potentials reported above were all measured in polar aprotic solvents (MeCN, DCM, or DMF). When measured in water, the *E*_1_ potentials experience an increase in the observed potential, by an average of +553 ± 26 mV (Table 2). This solvent dependence on redox potentials was previously noted for **4OAc-4py**, but its origin has not been discussed.^6,22^ Unfortunately, only a few complexes are water-soluble enough to be examined in this solvent, and *E*_2_ potentials were not observed in water since these oxidation events occur beyond the electrochemical window for water. We propose that the observed increases in redox potentials in water originate from hydrogen-bonding interactions between the cubane μ_3_-oxo ligands and water, which reduces the μ_3_-oxo donation to cobalt. The relative electronic effects of the cubane ligands appear to persist in water, however, as indicated by a similar *E*_1_/p*K*_a_ slope. The hydrogen-bonding effect is clearly demonstrated, even in aprotic solvent, by comparing **4pyrO-3pyrOH,py** with **4pyrO-4py**. The p*K*_a_ values of 2-hydroxypyridinium cation and pyridinium are essentially identical (5.23 and 5.25, respectively), and according to eqn (5), should give nearly identical *E*_1_ values for the corresponding cubanes. However, the observed *E*_1_ value for **4pyrO-3pyrOH,py** is 550 mV higher than that of **4pyrO-4py**. In the case of **2OAc,2Az-4AzH**, the experimental *E*_1_ value is 519 mV higher than predicted by eqn (5). The observed increases in redox potential with introduction of hydrogen bonding to the cubane (519 and 550 mV) are remarkably similar to that observed by changing the solvent to water (553 mV).

**Table 2:** Comparison of cubane redox potentials in aprotic solvent and water

Cubane	*E*_1_ (V), aprotic solvent	*E*_1_ (V), water^*a*^	Δ*E*_1_	Reference
**4OAc-4COOEtpy**	0.48	1.00	**0.52**	20 and 23
**NBA,3OAc-4py**	0.34	0.89	**0.55**	
**4OAc-4py**	0.28	0.85	**0.57**	
**4OAc-4Mepy**	0.27	0.84	**0.57**	20
**4OAc-4DMAP**	0.11	0.68	**0.57**	

^*a*^Potential *versus* Fc/Fc^+^.^24^

### DFT calculations of cubane electronic structures

To quantify the ligands' influence on the redox potentials of cubanes, an energy decomposition analysis (EDA) was performed. The results reveal that the X ligands stabilize the oxidized cubanes significantly better than L ligands (ESI and Table S1†). In **4OAc-4py**, the overall interaction energy between the [CoIII4O_4_]^4+^ ion and acetate is –158 kcal mol^–1^, while the energy between [CoIII4O_4_]^4+^ and pyridine is only –43 kcal mol^–1^. The interaction energies for the two ligand types increase upon oxidation to **[4OAc-4py]^+^**: –243 kcal mol^–1^ for acetate and –46 kcal mol^–1^ for pyridine. As can be seen, the [Co_4_O_4_]–acetate bonding is greatly strengthened upon oxidation by –85 kcal mol^–1^, but the pyridine–[Co_4_O_4_] bonding only slightly increases, by –2 kcal mol^–1^. The frozen electron density term, (FRZ) which describes electrostatic and steric contributions to bonding, is at least an order of magnitude larger for the acetate ligand, and doubles in magnitude upon oxidation. The polarization and charge transfer terms are also generally larger for acetate than for pyridine; however, they are less than an order of magnitude different. The size of the FRZ term indicates that electrostatic forces dominate the stability of the oxidized cubane. This finding seems intuitive as Co^III^ and Co^IV^ are both considered hard ions, and according to hard–soft acid–base theory should prefer ionic bonding interactions over dative interactions. To uncover trends in the bonding, EDA was also applied to **4TFA-4py** and **4OAc-4CNpy**. The energy difference in frozen components between **[4X-4L]^+^** and **4X-4L** (FRZ_ox_ – FRZ_red_ = ΔFRZ) for the [Co_4_O_4_]–X and [Co_4_O_4_]–L bonds plot against p*K*_a_ yields slopes of –0.8 kcal per (mol p*K*_a_) and –0.5 kcal per (mol p*K*_a_), respectively (Fig. S1†). Note that the energy values are within the error of DFT calculations, but there is a qualitative correlation between theory and the experimental redox trends.

**Fig. 6:**
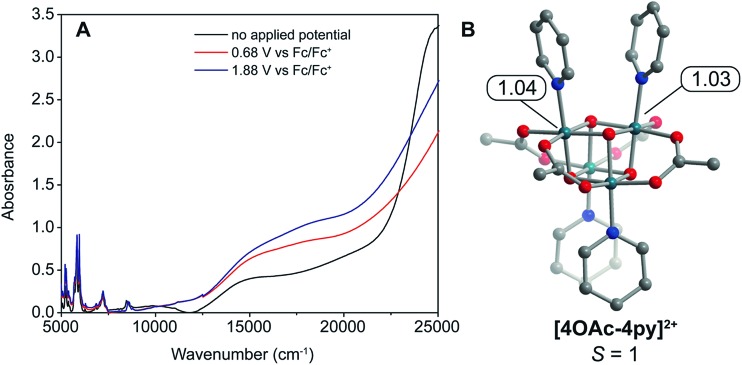
(A) UV-vis-NIR plot of **[4OAc-4py]**, **[4OAc-4py]^+^**, and **[4OAc-4py]^2+^** produced by applying no potential, 0.68 V, and 1.88 V, respectively, in an spectroelectrochemical cell (6 mM **[4OAc-4py]**, 1 mm path length, MeCN solvent, [^*n*^Bu_4_N]PF_6_ electrolyte, Pt mesh working electrode, Pt wire counter electrode, and Ag/AgNO_3_ nonaqueous reference electrode). (B) DFT-calculated structure of **[4OAc-4py]^2+^** with spin-densities indicated.

The doubly oxidized state, **[4OAc-4py]^2+^**, was analyzed by DFT calculations. These calculations determined that the *S* = 1 state is essentially degenerate with the *S* = 0 state (within 3.3 kcal mol^–1^). Interestingly, the calculations suggest two valence-trapped Co^IV^ centers, as opposed to a valence delocalized system. There have been conflicting reports of hole delocalization in cobalt cubane systems by DFT, and delocalization has been suggested as a product of a self-interaction error.^4,25^ In support of a localized valence, UV-vis-NIR spectroelectrochemistry measurements (Fig. 6) on **[4OAc-4py]**, **[4OAc-4py]^+^**, and **[4OAc-4py]^2+^** did not show any bands typical of a delocalized (Robin-Day class III) intervalence charge-transfer. However, there is a strong increase in absorption in the visible region (15 000–20 000 cm^–1^), and growth of a low intensity, broad band centered around 5000 cm^–1^ upon oxidation from **[4OAc-4py]** to **[4OAc-4py]^+^** to **[4OAc-4py]^2+^**; consistent with the Robin-Day Class II valence model. Recently, X-ray spectroscopic techniques provided additional support for a localized Co^IV^ center that undergoes very fast self-exchange with the other Co ions in the cubane **[4OAc-4py]^+^**.^13*a*^

### Implications for cubane intermediates with hydroxide and oxide ligands

Perhaps the most important utility of the energy relationships of eqn (5) and (6) is their ability to predict electronic properties for cubane-based intermediates that might be considered in mechanistic investigations. For example, these relationships allow thermodynamic evaluations of proposed hydroxyl- and oxo-intermediates in the recently proposed mechanism for cobalt cubane-mediated OER.^6^ It is important to emphasize that the redox values predicted by eqn (5) and (6) are under the strict assumption that fundamental bonding interactions between carboxylates, pyridonates, hydroxide, or oxide with Co_4_O_4_ are similar, in the sense that they are primarily electrostatic (*vide supra*). However, hydroxide and oxide ligands are stronger π-donors than carboxylates and pyridonates, and this may cause the true redox potentials of cubanes bound to hydroxide or oxide to deviate somewhat from those derived by eqn (5) and (6). Nevertheless, from simple ligand-field arguments, the stronger π-donating properties should actually destabilize more the lower oxidation states of cobalt, which should result in negative deviations from eqn (5) and (6).

A previous report from this laboratory described the aqueous behavior of Co_4_O_4_(OAc)_4_(py)_4_ (**4OAc-4py**) which reacts partially with hydroxide to produce [Co_4_O_4_(OAc)_3_(OH)_2_(py)_4_]^–^ (**[3OAc,2OH-4py]^–^**), and the latter species may be in equilibrium with Co_4_O_4_(OAc)_3_(OH)(H_2_O)(py)_4_ (**3OAc,OH-H_2_O,4py**) (eqn (7)).^6^ The bis(hydroxide) was characterized by NMR spectroscopy, and is proposed to be the resting state of the cubane during OER catalysis. However, as noted in this study, the formation of **[3OAc,2OH-4py]^–^** is low yielding since the equilibrium strongly favors **4OAc-4py**, which makes the isolation and direct electrochemical study of this species difficult.7
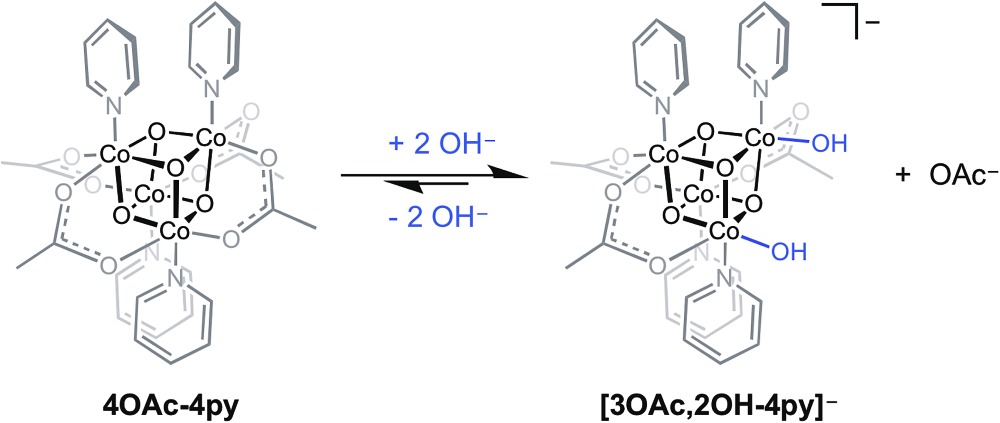



During catalysis, the Co(iii)-hydroxide moiety undergoes a series of proton- and electron-transfer steps to form a putative [Co_4_O_4_]^6+^ oxidation state, which was proposed on the basis of kinetic studies^6^ and published DFT^7^ calculations to be a Co^V^-oxo intermediate. Note that no spectroscopic or structural evidence of a Co^V^-oxo exists, and thus, any evidence supporting or discrediting its existence is valuable to understanding OER catalysis. The proposed mechanism invokes a key electron transfer from the Co^IV^-oxo to **[4OAc-4py]^+^** to generate the Co^V^-oxo intermediate ([Co_4_O_4_]^6+^ state) responsible for O–O bond formation.

No prior knowledge of the relative redox potentials contributed to the formulation of this disproportionation step; its existence is strongly inferred from the second-order rate dependence on **[4OAc-4py]^+^**. While this mechanistic study revealed that the [Co_4_O_4_]^6+^ state was achieved and only terminal oxygen ligands were involved, the level of water/hydroxide/oxo substitution and protonation states throughout the OER cycle are not known. For example, does the active species contain two terminal oxo-ligands, or only one, or none? The redox steps involved in this mechanism are undoubtedly coupled with proton transfers, as implicated by the first-order rate dependence on [OH^–^], but the ordering and extent of these processes are as yet unknown; in other words, kinetic data alone cannot definitively distinguish between a mechanism involving only aquo- and hydroxo-ligated cubanes and one involving the more unusual oxo ligand. Thus, if the redox potentials for corresponding oxo-, hydroxo-, and aquo-substituted Co_4_O_4_ clusters can be known (or well-estimated), it should be possible to rule out certain possible intermediates (those that cannot reach the [Co_4_O_4_]^6+^ state by disproportionation with **[4OAc-4py]^+^**), and discover which candidates are most plausible. The LFER equations (eqn (5) and (6)) allow simple evaluations of these candidate intermediates.

**Scheme 2:**
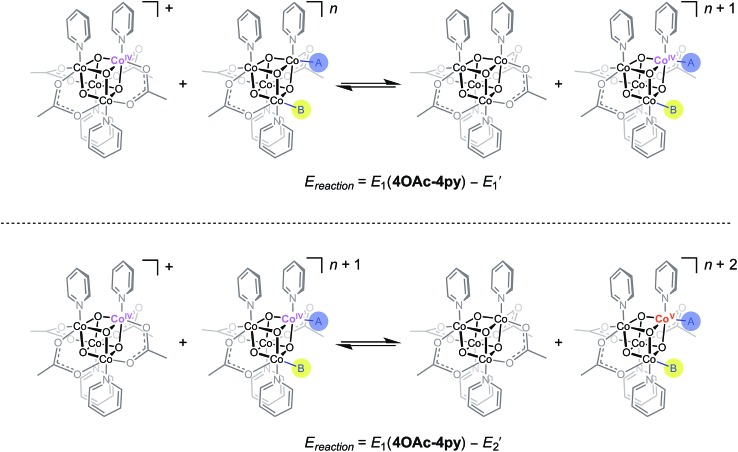
Redox disproportionation reactions with **[4OAc-4py]^+^**. A and B are either X or L ligands of the cubanes in Table 3.

Using the conjugate acid p*K*_a_ values of 15.7 for the OH^–^ ligand, and ∼36 for O^2–^,^18,26^ *E*_1_ and *E*_2_ redox potentials were calculated for the most likely catalytic intermediates (Scheme 2, Table 3). These *E*_1_ values suggest that oxidation of the catalytic intermediates in Table 3, from [CoIII4O_4_]^4+^ to [CoIII3Co^IV^O_4_]^5+^, by **[4OAc-4py]^+^** occurs spontaneously. The *E*_2_ potentials for the hydroxo-ligated cubanes are significantly higher than the *E*_1_ of **[4OAc-4py]^+^** which indicates that a simple electron transfer between cubanes to generate the [Co_4_O_4_]^6+^ state is not thermodynamically favored. However, if deprotonation of hydroxide to oxide occurred before electron-transfer, the oxidation to [Co_4_O_4_]^6+^ becomes favorable. Thus, these results suggest that the most likely active catalyst is **[3OAc,2OH-4py]^–^** (previously observed by NMR spectroscopy^6^), since it possesses redox couples consistent with cycling between [CoIII4O_4_]^4+^, [CoIII3Co^IV^O_4_]^5+^, and {[CoIII2CoIV2O_4_]^6+^ ↔ [CoIII3Co^V^O_4_]^6+^} states during OER catalysis. As previously reported,^6^ we favor the localized Co^V^ formalism when a strong σ and π donor such as O^2–^ is bound to a single Co ion, a viewpoint that is also supported by DFT. However, when multiple basic ligands (OH^–^ and O^2–^) are present, it is reasonable to expect the more delocalized states (*e.g.* CoIV2) to significantly contribute.

**Table 3:** Predicted redox potentials for hydroxo- and oxo-ligated cubanes

Cubane	*E*′_1_ ^*a*^ (V)	*E*′_2_ ^*a*^ (V)	[*E*_1_(4OAc-4py) – *E*′_1_] (V)	[*E*_1_(4OAc-4py) – *E*′_2_] (V)
**[4OAc,OH-4py]^–^**	–0.09	+0.86	+0.37	–0.58
**[4OAc,O-4py]^2–^**	–0.70	+0.21	+0.96	–0.07
**3OAc,OH-H_2_O,4py**	0.01	0.96	+0.27	–0.69
**[3OAc,2OH-4py]^–^**	–0.41	+0.51	+0.69	–0.23
**[3OAc,OH,O-4py]^2–^**	–1.02	–0.14	+1.30	+0.42
**[3OAc,2O-4py]^3–^**	–1.63	–0.78	+1.91	+1.06

^*a*^Potential *versus* Fc/Fc^+^ in polar aprotic solvent.

**Scheme 3:**
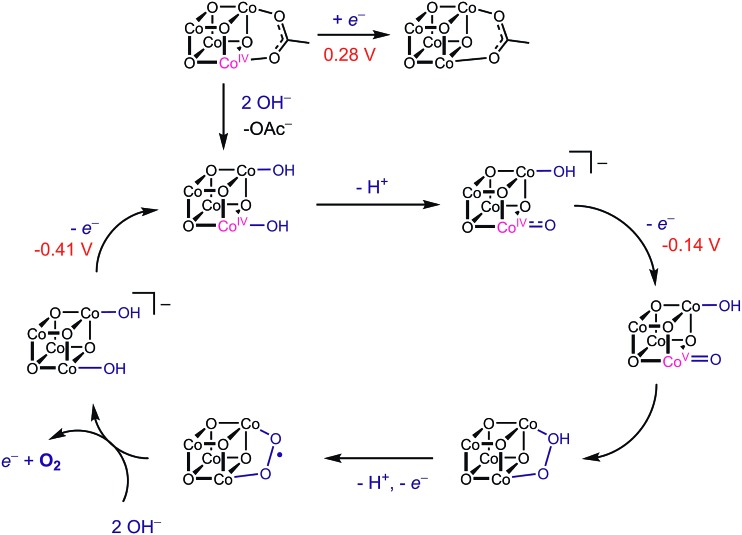
Proposed mechanism of OER by Co_4_O_4_ with redox potentials (*vs.* Fc/Fc^+^) for each oxidation step shown.

With this information, a revised OER catalytic cycle can be proposed (Scheme 3). This updated mechanism allows for two kinetically indistinguishable O–O coupling pathways, an acid–base mechanism or a radical-coupling pathway. Recently, Nocera and coworkers obtained direct evidence for the coupling of adjacent cobalt-oxygen species at pH 7 in the CoP_i_ catalyst, and several DFT calculations have shown this pathway to be energetically reasonable.^4,27,28^ Nonetheless, these predicted redox values show that cubanes with hydroxo- and oxo-ligands can reach the a formal CoIV2 or Co^V^ oxidation state by redox disproportionation reactions.

## Conclusions

Cobalt oxo cubane complexes are readily manipulated by convenient syntheses that allow exchange of the nitrogen-donor and carboxylate ligands, the latter in a sequential, selective manner. This synthetic chemistry allows tuning of the cubane electronic properties over a wide range. Primary variables that influence the cubane's redox properties are the donor properties of the anionic and neutral ligands, and the incorporation of hydrogen-bond interactions. Two important principles that result from this work, that should guide OER catalyst design, are that (1) the [Co_4_O_4_] cubane is predictably modifiable over a remarkable range of electrochemical and chemical properties, and (2) the accessibility of a formal CoIV2/Co^V^-oxo intermediate by the cubane is energetically feasible given the appropriate ligand set. The strong influence of hydrogen bonding suggests that catalyst design must not only address the primary coordination sphere, but also second-sphere interactions between the catalytic core and the reaction medium. The fundamental reaction chemistry developed in this study provides new strategies for functionalization of the cubane with a vast array of chelating ligands and N-heterocyclic donors, and should allow incorporation of catalytic cubanes into more complex architectures involving biological molecules, surfaces, and 3-dimensional networks. Importantly, the linear free energy relationships described above are useful in predicting the redox properties of newly designed cubane-based materials.

## Conflict of interest

No competing financial interests have been declared.

## References

[cit1] Krewald V., Retegan M., Cox N., Messinger J., Lubitz W., DeBeer S., Neese F., Pantazis D. A. (2015). Chem. Sci..

[cit2] Fillol J. L., Codolà Z., Garcia-Bosch I., Gómez L., Pla J. J., Costas M. (2011). Nat. Chem..

[cit3] McAlpin J. G., Surendranath Y., Dincǎ M., Stich T. A., Stoian S. A., Casey W. H., Nocera D. G., Britt R. D. (2010). J. Am. Chem. Soc..

[cit4] Li X., Siegbahn P. E. M. (2013). J. Am. Chem. Soc..

[cit5] Fernando A., Aikens C. M. (2015). J. Phys. Chem. C.

[cit6] Nguyen A. I., Ziegler M. S., Oña-Burgos P., Sturzbecher-Hohne M., Kim W., Bellone D. E., Tilley T. D. (2015). J. Am. Chem. Soc..

[cit7] Su X.-J., Gao M., Jiao L., Liao R.-Z., Siegbahn P. E. M., Cheng J.-P., Zhang M.-T. (2015). Angew. Chem., Int. Ed..

[cit8] Liu F., Concepcion J. J., Jurss J. W., Cardolaccia T., Templeton J. L., Meyer T. J. (2008). Inorg. Chem..

[cit9] Gerken J. B., McAlpin J. G., Chen J. Y. C., Rigsby M. L., Casey W. H., Britt R. D., Stahl S. S. (2011). J. Am. Chem. Soc..

[cit10] Winkler J. R. and Gray H. B., in Molecular Electronic Structures of Transition Metal Complexes I, Structure and Bonding, ed. D. M. P. Mingos, P. Day and J. P. Dahl, Springer Berlin Heidelberg, 2011, pp. 17–28.

[cit11] Costentin C., Porter T. R., Savéant J.-M. (2016). J. Am. Chem. Soc..

[cit12] Nguyen A. I., Hadt R. G., Solomon E. I., Tilley T. D. (2014). Chem. Sci..

[cit13] Hadt R. G., Hayes D., Brodsky C. N., Ullman A. M., Casa D. M., Upton M. H., Nocera D. G., Chen L. X. (2016). J. Am. Chem. Soc..

[cit14] Beattie J. K., Hambley T. W., Klepetko J. A., Masters A. F., Turner P. (1998). Polyhedron.

[cit15] Zhou X.-X., Welch C. J., Chattopadhyaya J. (1986). Acta Chem. Scand., Ser. B.

[cit16] Dufour N., Lebuis A.-M., Corbeil M.-C., Beauchamp A. L., Dufour P., Dartiguenave Y., Dartiguenave M. (1992). Can. J. Chem..

[cit17] Jeffrey G. A., An Introduction to Hydrogen Bonding, Oxford University Press, 1997.

[cit18] Handbook of Chemistry and Physics, CRC Press, Florida, 87th edn, 2006.

[cit19] Smith R. M. and Motekaitis R. J., NIST Standard Reference Database 46, 3.0, National Institute of Standards and Technology, Washington, D.C., 1997.

[cit20] Berardi S., La Ganga G., Natali M., Bazzan I., Puntoriero F., Sartorel A., Scandola F., Campagna S., Bonchio M. (2012). J. Am. Chem. Soc..

[cit21] Dimitrou K., Brown A. D., Concolino T. E., Rheingold A. L., Christou G. (2001). Chem. Commun..

[cit22] McCool N. S., Robinson D. M., Sheats J. E., Dismukes G. C. (2011). J. Am. Chem. Soc..

[cit23] Ullman A. M., Liu Y., Huynh M., Bediako D. K., Wang H., Anderson B. L., Powers D. C., Breen J. J., Abruña H. D., Nocera D. G. (2014). J. Am. Chem. Soc..

[cit24] Georges J., Desmettre S. (1984). Electrochim.
Acta.

[cit25] McAlpin J. G., Stich T. A., Ohlin C. A., Surendranath Y., Nocera D. G., Casey W. H., Britt R. D. (2011). J. Am. Chem. Soc..

[cit26] Cotton F. A., Wilkinson G., Murillo C. A. and Bochmann M., Advanced Inorganic Chemistry, John Wiley & Sons, 6th edn, 1999.

[cit27] Ullman A. M., Brodsky C. N., Li N., Zheng S.-L., Nocera D. G. (2016). J. Am. Chem. Soc..

[cit28] Wang L.-P., Van Voorhis T. (2011). J. Phys. Chem. Lett..

