# Supplementary material for: Synthetic control and empirical prediction of redox potentials for Co4O4 cubanes over a 1.4 V range: implications for catalyst design and evaluation of high-valent intermediates in water oxidation
Source: Chem Sci. 2017 Apr 7;8(6):4274–84. doi: 10.1039/c7sc00627f (PMC5635813; doi:10.1039/c7sc00627f)
Supplement: Supplementary file 1 [file SC-008-C7SC00627F-s001.pdf]

Electronic Supplementary Material (ESI) for Chemical Science.
This journal is © The Royal Society of Chemistry 2017

Supporting Information for:

Synthetic Control and Empirical Prediction of Redox Potentials for Co_4O_4 Cubanes over a 1.4 V Range: Implications for Catalyst Design and Evaluation of High-Valent Intermediates in Water Oxidation

Andy I. Nguyen, Jianing Wang, Daniel S. Levine, Micah S. Ziegler, T. Don Tilley*

General considerations. Cobalt(II) nitrate hexahydrate, pyridine, hydrogen peroxide (34-37% in water), 4-methoxypyridine, 4-cyanopyridine, 4-*N*,*N*-dimethylaminopyridine, 4-nitrobenzoic acid, chloroacetic acid, 2-hydroxypyridine, and 7-azaindole were purchased from Sigma-Aldrich and used without further purification. Sodium acetate trihydrate was purchased from EMD. Solvents were purchased from Fisher-Scientific and used without any further purification. The complexes $Co_4O_4(OAc)_4(py)_4$[1] and $Co_4O_4(OAc)_4$(4-cyanopyridine)$_4$[1] were synthesized according to published procedures. The synthetic procedures described below were conducted in air.

Physical methods. Routine NMR spectra were recorded on Bruker AV-400 spectrometers at room temperature. CD_2Cl_2 and acetonitrile-d_3 were purchased from Cambridge Isotope Laboratories. ^{1}H NMR spectra were referenced to residual protio-solvent peaks (δ 5.32 for CD_2Cl_2, δ 1.94 for acetonitrile-d_3). Elemental analyses were carried out by the College of Chemistry Microanalytical Laboratory at the University of California, Berkeley. After purification by column chromatography, the products sometimes possessed solvent that is difficult to remove under vacuum, as determined by NMR spectroscopy. Thus, some of the calculated compositions include solvent of crystallization.

Electrochemical measurements were collected with a three-electrode setup on a BASi Epsilon potentiostat (glassy carbon working electrode, Pt wire auxiliary electrode, and Ag wire floating reference). Ferrocene was added as an internal standard.

UV-Visible-NIR spectra were recorded on a Varian Cary 5000 using Cary WinUV software (v. 3.00(339)) and employing a spectroelectrochemical cell containing a Pt-mesh working electrode, a Pt-wire auxiliary electrode, and a non-aqueous, quasi-reference $Ag/AgNO_3$ electrode (0.1 M [$^n Bu_4N$][PF_6] with $AgNO_3$ (0.01 M) in acetonitrile).

$Co_4O_4(OAc)_4$(4-methoxypyridine). 4-Methoxypyridine (0.150 g, 1.42 mmol) was added to a 10 mL MeCN solution of $Co_4O_4(OAc)_4(py)_4$ (0.100 g, 0.118 mmol), and the resulting solution was heated to 90 °C for 16 hours, yielding a dark solution. Volatile compounds were removed *in vacuo*, and the resulting solid was washed with Et_2O (3 x 10 mL) to give the product (0.113 g, 100%). The ^{1}H NMR spectrum is consistent with that reported.[2]

$Co_4O_4(OAc)_4$(4-*N*,*N*-dimethylaminopyridine)$_4$. *N*,*N*-Dimethylaminopyridine (0.860 g, 7.0 mmol) was added to a 30 mL MeCN solution of $Co_4O_4(OAc)_4(py)_4$ (0.500 g, 0.59

mmol), and the resulting solution was heated to 80°C for 16 hours, yielding a dark solution. The solution was dried *in vacuo*, and the solid was washed with Et_2O (3 x 100 mL). The resulting solid was recrystallized from hot MeCN (~10 mL) to yield 0.400 g (67%) of dark solid. The complex was further purified by recrystallization, by allowing layered hexane to diffuse into a dichloromethane solution of the complex. ^{1}H NMR (400 MHz, CD_2Cl_2): 8.19 (d, 8H, aryl-*H*), 7.01 (d, 8H, aryl-*H*), 3.57 (s, 24H, –C*H₃*), 2.59 (s, 12H, –C*H₃*). Anal. Calcd, for $C_{36}H_{52}Co_4N_8O_{12}{\cdot}0.45\ CH_2Cl_2$: C, 41.19, H, 5.02; N, 10.54. Found: C, 41.03; H, 5.20; N, 10.58.

$Co_4O_4(O_2CH_2Cl)_4(py)_4$. Chloroacetic acid (0.049 g, 0.96 mmol) was added to a 5 mL solution of $Co_4O_4(OAc)_4(py)_4$ (0.100 g, 0.125 mmol) in MeCN, and the resulting solution was heated to 60°C for 16 hours, to give a dark solution. The solution was dried *in vacuo*, and the solid was recrystallized by layer diffusion of hexanes into a dichloromethane solution to yield 0.081 g (70%) of a dark solid. ^{1}H NMR (300 MHz, $CDCl_3$): 8.58 (d, 8H, aryl-*H*), 7.54 (t, 4H aryl-*H*), 7.09 (t, 8H aryl-*H*), 4.10 (s, 8H, –CH_2). Anal. Calcd. for $C_{24}H_{28}Cl_4Co_4N_4O_4{\cdot}0.83\ CH_2Cl_2$: C, 33.71; H, 3.38; N, 6.33. Found: C, 34.20; H, 3.14; N, 5.84.

$Co_4O_4(O_2CCF_3)_4(py)_4$. Trifluoroacetic acid (0.110 g, 0.96 mmol) was added to a 5 mL solution of $Co_4O_4(OAc)_4(py)_4$ (0.200 g, 0.23 mmol) in MeCN, and the resulting solution was heated to 70°C for 16 hours, to give a dark solution and a black crystalline solid. The solid product was collected by decantation, and was washed with Et_2O (3 x 10 mL) to give 0.192 g of the product (77%). Anal. Calcd. for $C_{28}H_{20}Co_4F_{12}N_4O_{12}$: C, 31.48; H, 1.89; N, 5.25. Found: C, 31.62; H, 1.97; N, 5.40.

$Co_4O_4(OAc)_3(O_2CCF_3)(py)_4$, $Co_4O_4(OAc)_2(O_2CCF_3)_2(py)_4$, $Co_4O_4(OAc)(O_2CCF_3)_3(py)_4$.

Trifluoroacetic acid (0.0334 g, 0.59 mmol) was added to a 10 mL solution of $Co_4O_4(OAc)_4(py)_4$ (0.25 g, 0.29 mmol) in MeCN, and the resulting solution was heated to 80°C for 16 hours, to give a dark solution. Analysis by TLC with 2:1 hexanes/acetone gave 4 spots with R_f values of 0.8, 0.5, 0.2, and 0. Each species was isolated by silica gel column chromatography (eluted with acetone and hexanes gradient), and the fractions containing the pure compound were evaporated *in vacuo*.

For $Co_4O_4(OAc)_3(O_2CCF_3)(py)_4$: yield 0.050 g, 19% (with respect to $Co_4O_4(OAc)_4(py)_4$). Anal. Calcd. for $C_{28}H_{29}Co_4F_3N_4O_{12}$: C, 37.11; H, 3.23; N, 6.18. Found: C, 37.30; H, 3.52; N, 5.89. ^{1}H NMR (400 MHz, CD_2Cl_2): δ 8.46 (m, 8H, aryl-*H*), 7.56 (t, 4H, aryl-*H*), 7.08 (t, 8H, aryl-*H*), 2.16 (s, 6H, –CH_3), 2.09 (s, 3H, –CH_3).

For $Co_4O_4(OAc)_2(O_2CCF_3)_2(py)_4$: yield 0.042 g, 15.2% (with respect to $Co_4O_4(OAc)_4(py)_4$). Anal. Calcd. for $C_{28}H_{26}Co_4F_6N_4O_{12}{\cdot}C_3H_6O$: C, 36.56; H, 3.17; N, 5.50. Found: C, 36.20 H, 3.12; N, 5.49. ^{1}H NMR (400 MHz, CD_2Cl_2): δ 8.36 (m, 8H, aryl-*H*), 7.50 (t, 4H, aryl-*H*), 7.01 (t, 8H aryl-*H*), 2.07 (s, 6H, –CH_3).

For $Co_4O_4(OAc)(O_2CCF_3)_3(py)_4$: yield 0.033 g, 11.4% (with respect to $Co_4O_4(OAc)_4(py)_4$). Anal. Calcd. for $C_{28}H_{23}Co_4F_9N_4O_{12}{\cdot}C_3H_6O$: C, 34.72; H, 2.73; N, 5.22. Found: C, 34.46; H, 2.59; N, 5.02. ^{1}H NMR (400 MHz, CD_2Cl_2): δ 8.39 (m, 8H, aryl-*H*), 7.60 (t, 4H, aryl-*H*), 7.13 (t, 8H, aryl-*H*), 2.22 (s, 3H, –CH_3).

$Co_4O_4(O_2CCF_3)_4$(4-methoxypyridine)$_4$. Trifluoroacetic acid (0.028 g, 0.24 mmol) was added to a 5 mL solution of $Co_4O_4(OAc)_4$(4-methoxypyridine)$_4$(py)$_4$ (0.0575 g, 0.067 mmol) in MeCN, and the resulting solution was heated to 70°C for 16 hours, to give a dark solution. The solution was evaporated to dryness *in vacuo*, and the resulting solid was dissolved in a minimum amount of dichloromethane (ca. 1 mL) and purified by silica chromatography with 6:4 hexanes:acetone eluant. Anal. Calcd. for $C_{32}H_{28}Co_4F_{12}N_4O_{16}\cdot C_3H_6O$: C, 33.73; H, 2.75; N, 4.50. Found: C, 33.24; H, 2.77; N, 4.60. ^{1}H NMR (400 MHz, CD_3CN): 8.08 (d, 8H, aryl-*H*), 6.83 (d, 8H, aryl-*H*), 3.89 (s, 12H, –CH_3).

$Co_4O_4(O_2CCF_3)_4$(4-cyanopyridine)$_4$. Trifluoroacetic acid (0.245 g, 2.15 mmol) was added to a 10 mL solution of $Co_4O_4(OAc)_4(py)_4$ (0.500 g, 0.587 mmol) in MeCN, and the resulting solution was heated to 70°C for 16 hours, to give a dark solution. The solution was dry-loaded onto silica, and then purified by column chromatography using a 7:3 hexanes:acetone eluant. The solid was by precipitated from MeCN (ca. 2 mL) with excess Et_2O (0.164 g, 24%). Anal. Calcd. for $C_{32}H_{16}Co_4F_{12}N_8O_{12}\cdot 2\ H_2O$: C, 31.92; H, 1.67; N, 9.30. Found: C, 31.6; H, 1.57; N, 9.12. ^{1}H NMR (400 MHz, CD_3CN): δ 8.56 (d, 8H, aryl-*H*), 7.64 (d, 4H, aryl-*H*), 2.16 (s, 4H, H_2O).

$Co_4O_4(OAc)_3$(4-nitrobenzoate)(py)$_4$, $Co_4O_4(OAc)_2$(4-nitrobenzoate)$_2$(py)$_4$, Co_4O_4(OAc)(4-nitrobenzoate)$_3$(py)$_4$, Co_4O_4(4-nitrobenzoate)$_4$(py)$_4$.
4-Nitrobenzoic acid (0.029 g, 0.17 mmol) was added to a 10 mL solution of $Co_4O_4(OAc)_4(py)_4$ (0.073 g, 0.086 mmol) in MeCN, and the resulting solution was heated to 80°C for 16 hours to give a dark orange solution. Analysis by TLC with 2:1 hexanes/acetone gave 5 spots with R_f values of 0.9, 0.7, 0.5, 0.2 and 0. Silica gel column chromatography was performed to isolate the above species, and the fractions containing the pure compound were evaporated to dryness *in vacuo*. Each compound was recrystallized by layering hexanes onto a dichloromethane solution.

Yields (with respect to $Co_4O_4(OAc)_4(py)_4$):
For $Co_4O_4(OAc)_3$(4-nitrobenzoate)py$_4$: yield 0.012 g, 15%.^{1}H NMR (400 MHz, CD_2Cl_2): δ 8.51 (m, 8H, aryl-*H*), 8.11 (m, 4H, aryl-*H*), 7.56 (m, 4H, aryl-*H*), 7.10 (m, 8H, aryl-*H*), 2.14 (s, 3H, –CH_3), 2.09 (s, 6H, –CH_3).
For $Co_4O_4(OAc)_2$(4-nitrobenzoate)$_2$(py)$_4$: yield: 0.007 g, 7.7%. Anal. Calcd. for $C_{43}H_{35}Co_4N_7O_{18}\cdot 0.3\cdot CH_2Cl_2$: C, 43.38; H, 2.99; N, 8.18. Found: C, 43.16; H, 3.23; N, 7.94. ^{1}H NMR (400 MHz, CD_2Cl_2): δ 8.47 (d, 8H, aryl-*H*), 8.13 (d, 4H, aryl-*H*), 7.99 (d, 4H, aryl-*H*), 7.63 (m, 4H, aryl-*H*), 7.13 (d, 8H, aryl-*H*), 2.11 (s, 6H, –CH_3).
For Co_4O_4(OAc)(4-nitrobenzoate)$_3$(py)$_4$: yield 0.017 g, 17%. ^{1}H NMR (400 MHz, CD_2Cl_2): δ 8.59 (d, 8H, aryl-*H*), 8.13 (m, 12H, aryl-*H*), 7.63 (t, 4H, aryl-*H*), 7.14 (t, 8H, aryl-*H*), 2.17 (s, 3H, –CH_3).
For Co_4O_4(4-nitrobenzoate)$_4$(py)$_4$: yield 0.005 g, 4.6%. Anal. Calcd. for $C_{48}H_{36}Co_4N_8O_{20}\cdot 1.2\ CH_2Cl_2$: C, 42.74; H, 2.80; N, 8.11. Found: C, 43.08; H, 2.86; N, 7.76. ^{1}H NMR (400 MHz, CD_2Cl_2): δ 8.64 (d, 8H, aryl-*H*), 8.17 (m, 16H, aryl-*H*), 7.67 (t, 4H, aryl-*H*), 7.17 (t, 8H, aryl-*H*).

Co_4O_4(2-pyridonate)$_4$(2-hydroxypyridine)$_3$(py). 2-Hydroxypyridine (0.268 g, 2.81 mmol) was added to a 10 mL solution of $Co_4O_4(OAc)_4(py)_4$ (0.2 g, 0.24 mmol) in MeCN, and the resulting solution was heated at 80°C for 16 hours, to give a dark solution with a black crystalline solid. The solid was collected by filtration, and analysis by TLC with 60/40 hexanes/acetone shows three spots with R_f value of around 0.8, 0.7, 0.6. Silica gel column chromatography was performed to isolate the product (R_f = 0.8). Yield: 0.047 g, 37%. HR-ESI-MS: *m/z* = 1040.99 ($[Co_4O_4(C_5H_4NO)_4(C_5H_5NO)_3(C_5H_5N)H]^+$). Anal. Calcd. for $C_{40}H_{36}Co_4N_8O_{11}$: C, 46.17; H, 3.49; N, 10.77. Found: C, 46.09; H, 3.75; N, 10.78. ^{1}H NMR (400 MHz, CD_2Cl_2): δ 8.79 (d, 1H, aryl-*H*), 8.16 (m, 2H, aryl-*H*), 8.00 (dd, 1H, aryl-*H*), 7.94 (dd, 1H, aryl-*H*), 7.43 (td, 1H, aryl-*H*), 7.09-7.30 (m, 13H, aryl-*H*), 6.59-6.72 (m, 8H, aryl-*H*), 6.25 (m, 3H, aryl-*H*), 6.16 (td, 1H, aryl-*H*), 5.74 (m, 2H, aryl-*H*), 5.57 (dd, 1H, aryl–*H*).

Co_4O_4(2-pyridonate)$_4$(4-*N*,*N*-dimethylaminopyridine)$_4$. 4-*N*,*N*-Dimethylaminopyridine (1.287 g, 10.5 mmol) was added to a 10 mL suspension of Co_4O_4(2-pyridonate)$_4$(2-hydroxypyridine)$_3$(py) (0.15 g, 0.13 mmol) in MeCN, and the resulting solution was heated to 90°C for 16 hours, to give a dark solution with a solid precipitate. The solution was decanted and solids were heated with acetonitrile (10 mL) at 80°C for 15 minutes, then washed with acetonitrile (3 x 10). Recrystallized by layer diffusion of hexanes into a dichloromethane solution yielded 0.081 g (49%). Anal. Calcd. for $C_{48}H_{56}Co_4N_{12}O_8{\cdot}3H_2O$: C, 47.3; H, 5.13; N, 13.79. Found: C, 47.03; H, 5.35; N, 13.49. ^{1}H NMR (400 MHz, CD_2Cl_2): δ 8.20 (d, 4H, aryl-*H*), 7.56 (d, 4H, aryl-*H*), 7.21 (dd, 4H, aryl-*H*), 7.12 (td, 4H, aryl-*H*), 6.42 (dd, 4H, aryl-*H*), 6.35 (dd, 4H, aryl-*H*), 6.05 (td, 4H, aryl-*H*), 5.78 (dd, 4H, aryl-*H*), 2.88 (s, 24H, –CH_3).

Co_4O_4(2-pyridonate)$_4$(py)$_4$. Pyridine (1.157 g, 1.18 mL, 14.6 mmol) was added to a 10 mL suspension of Co_4O_4(2-pyridonate)$_4$(2-hydroxypyridine)$_3$(py) (0.386 g, 0.034 mmol) in MeCN, and the resulting solution was heated to 80°C for 16 hours, to give a dark solution with a black solid. The solid was collected and recrystallized by layer diffusion of hexanes into a dichloromethane solution. Yield: 0.018 g, 45%. Anal. Calcd. for $C_{40}H_{36}Co_4N_8O_8{\cdot}CH_2Cl_2$: C, 45.71; H, 3.55; N, 10.40. Found: C, 46.09; H, 3.74; N, 10.78. ^{1}H NMR (400 MHz, CD_2Cl_2): δ 8.74 (d, 4H, aryl-*H*), 8.10 (d, 4H, aryl-*H*), 7.37 (m, 4H, aryl-*H*), 7.16 (m, 4H, aryl-*H*), 7.08 (m, 8H, aryl-*H*), 6.55 (t, 4H, aryl-*H*), 6.50 (d, 4H, aryl-*H*), 6.06 (t, 4H, aryl-*H*).

$Co_4O_4(OAc)_2$(7-azin)$_2$(7-azaindole)$_4$. 7-Azaindole (0.078 g, 0.66 mmol) was added to a 10 mL solution of $Co_4O_4(OAc)_4py_4$ (0.047 g, 0.055 mmol) in MeCN, and the resulting solution was heated at 80°C for 16 hours, to give a dark solution with a black crystalline precipitate (single-crystal XRD quality). The solid was collected by filtration. Yield: 0.030 g, 48%. Anal. Calcd for $C_{46}H_{40}Co_4N_{12}O_8{\cdot}0.55NCCH_3$: C, 49.31; H, 3.66; N, 15.32. Found: C, 49.37; H, 3.51; N, 15.47.

Figure S1. Differential pulse voltammetry of cubane complexes with irreversible E_1 and/or E_2

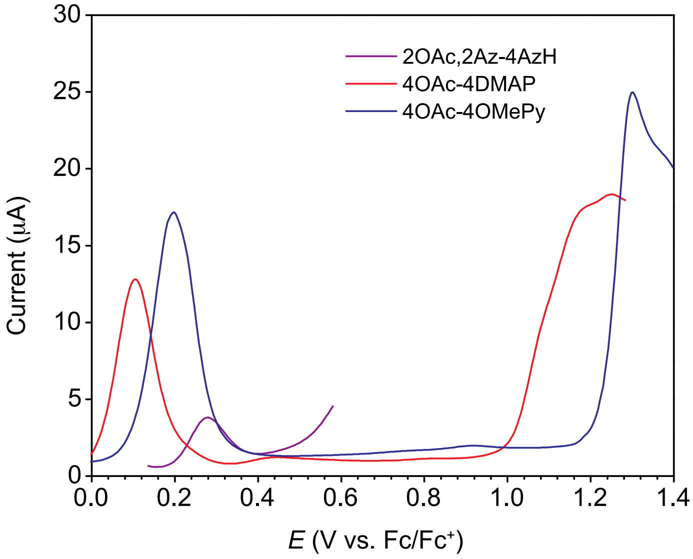

Computational details. All calculations were carried out using the Q-Chem 4.2.2 software package.[3] Geometry optimizations and EDA calculations for LFER analysis were carried out using the B3PW91-D3 functional evaluated on a (99,590) grid.[4] The 6-31G* basis set was used for light atoms (CHNOF), while the LanL2DZ ECP and basis set were used for Co. Geometry optimizations and spin-density calculations for the cobalt cubane dication were carried out both at the B3PW91-D3 level and at the more sophisticated ωB97M-V level.[5] These calculations employed the 6-31+G** basis set for light atoms (CHNO) and either the 6-31+G** all-electron basis or the relativistic small-core ECP of the Stuttgart group for Co.[6] All combinations of basis sets and functionals produce qualitatively similar results.

Table S1. Summary of EDA results

Cubane Fragments	Total Energy (Hatree)	Frozen (FRZ) term (kJ/mol)	Polarization (POL) term (kJ/mol)	Charge transfer (P-DEL) term (kJ/mol)
$[Co_4O_4(py)_4]^{4+}$ & $[4OAc]^{4-}$	-2788.04984	-250.3	-179.8	-232.2
$[Co_4O_4(py)_4]^{5+}$ & $[4OAc]^{4-}$	-2787.819219	-504.3	-223.2	-287.2
$[Co_4O_4(OAc)_4]$ & [4py]	-2788.04984	-0.9	-44.9	-137.1
$[Co_4O_4(OAc)_4]^{+}$ & [4py]	-2787.819219	-16.6	-49.4	-125.3
$[Co_4O_4(OAc)_4]$ & [4CNpy]	-3156.839256	2.7	-44.9	-143.1
$[Co_4O_4(OAc)_4]^{+}$ & [4CNpy]	-3156.581096	-5.4	-59.3	-138.8
$[Co_4O_4(py)_4]^{4+}$ & $[4TFA]^{4-}$	-3978.470358	-275.6	-160.3	-223.0
$[Co_4O_4(py)_4]^{5+}$ & $[4TFA]^{4-}$	-3978.202673	-514.7	-202.0	-270.9

Figure S2. Plot of FRZ energies differences as a function of ligand basicity.

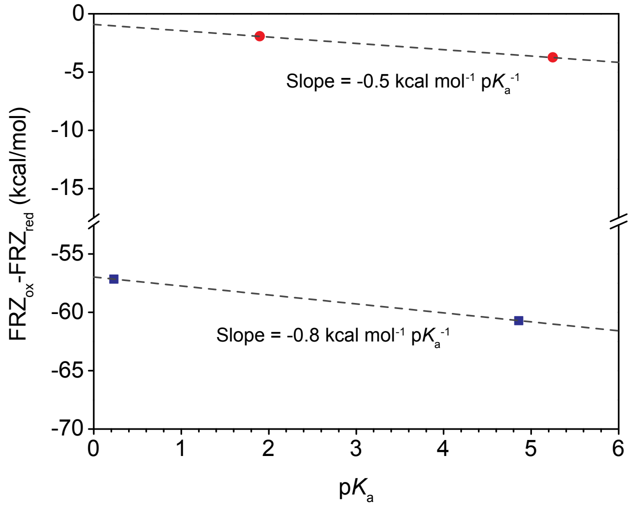

Coordinates and energy for $[4OAc\text{-}4py]^{+}$

```
1 2
--
2 2
C         -1.21951       -1.64414        2.81484
C         -1.67858       -2.32622        4.07952
H         -1.39513       -1.70161        4.93415
H         -1.17429       -3.28946        4.19117
H         -2.76264       -2.45199        4.07710
C          1.22812        1.71290       -2.72588
C          1.82312        2.42781       -3.91430
H          2.83059        2.03840       -4.09492
H          1.86580        3.50378       -3.73998
H          1.22834        2.21375       -4.80754
C          2.13465       -2.63597       -0.71021
C          3.11789       -3.74801       -0.97978
H          4.09463       -3.29774       -1.19353
H          2.81168       -4.31565       -1.86119
H          3.21596       -4.40059       -0.11022
C          1.94884        0.51226        3.36131
```

H	0.88826	0.71116	3.46389
C	2.90188	1.00577	4.24213
H	2.58865	1.60618	5.08949
C	4.24474	0.71600	4.01061
H	5.01224	1.08351	4.68574
C	4.58763	-0.05060	2.89939
H	5.62047	-0.29933	2.67889
C	3.57765	-0.50359	2.06050
H	3.77111	-1.10580	1.18223
C	1.22273	3.44576	1.95760
H	0.15409	3.49473	2.12506
C	2.13287	4.17182	2.71475
H	1.77657	4.81740	3.51094
C	3.49015	4.05219	2.42673
H	4.22689	4.60854	2.99919
C	3.88774	3.20839	1.39169
H	4.93408	3.08178	1.13505
C	2.91573	2.52137	0.67640
H	3.15088	1.83854	-0.13203
C	-3.64859	-2.07038	-0.13359
H	-3.19515	-2.54427	0.72776
C	-4.72449	-2.62434	-0.81483
H	-5.14022	-3.57119	-0.48600
C	-5.24528	-1.94394	-1.91286
H	-6.08545	-2.35289	-2.46714
C	-4.67292	-0.73086	-2.28972
H	-5.04466	-0.17074	-3.14120
C	-3.60300	-0.23665	-1.55564
H	-3.09055	0.68659	-1.79932
C	-1.33561	-2.74089	-2.46419
H	-0.98127	-3.16332	-1.53094
C	-2.19947	-3.41429	-3.31806
H	-2.53523	-4.41394	-3.06348
C	-2.61256	-2.78546	-4.49047
H	-3.28040	-3.29143	-5.18203
C	-2.15472	-1.49848	-4.76220
H	-2.45103	-0.97062	-5.66279
C	-1.29937	-0.88563	-3.85581
H	-0.90420	0.11079	-4.00418
N	2.29069	-0.22658	2.29908
N	1.61353	2.64316	0.96038
N	-3.10872	-0.90441	-0.50583
N	-0.90097	-1.50502	-2.73786
O	-0.44682	-1.50154	0.00207
O	-0.19966	0.61442	1.39328
O	1.48470	0.16476	-0.28029
O	-0.92018	0.65736	-1.06260
O	0.04023	-1.71069	2.59627
O	-2.09711	-1.06910	2.11382
O	0.97290	2.41773	-1.70571
O	1.05379	0.45934	-2.87650
O	1.58376	-2.10268	-1.70972
O	1.98610	-2.32427	0.52367
Co	0.85335	-0.85848	1.07493
Co	-1.63139	-0.13347	0.45898
Co	0.31174	1.62735	-0.08680
Co	0.30387	-0.64861	-1.50127

--
-1 1

O	-2.67843	1.43373	0.80575
O	-1.05473	2.93492	0.24133
C	-2.22446	2.60304	0.61092
C	-3.19110	3.73112	0.87601
H	-3.38141	3.78617	1.95362
H	-4.14657	3.51865	0.38840
H	-2.78728	4.68213	0.52555

Final Energy: -2787.8089877182

Coordinates and energy for 4OAc-4py

0 1
--
1 1

Co	0.19544	-0.07776	9.29757
Co	0.13012	0.07006	6.50200
Co	1.97072	1.38309	7.93676
O	1.97231	0.04700	6.63751
O	2.02803	-0.07796	9.07421
N	3.92204	1.58224	7.93032
O	1.81554	2.73884	6.53311
O	0.13486	1.21354	7.95789
O	0.29591	1.58487	5.27453
N	-1.74589	-0.04956	9.53768
N	-1.82134	0.06190	6.33769
C	-2.49881	1.13739	5.92401
H	-1.89030	2.00584	5.70070
C	1.07027	2.55896	5.52658
C	6.67306	1.88230	7.85753
H	7.75313	2.00131	7.82769
C	-2.40886	1.07483	9.24402
H	-1.78591	1.88117	8.87047
C	5.96731	2.11041	9.03644
H	6.47159	2.41089	9.94974
C	4.59094	1.35493	6.79561
H	3.97254	1.03075	5.96458
C	4.58715	1.94577	9.03221
H	3.96872	2.11029	9.90622
C	1.13471	3.63570	4.46034
H	1.27268	4.61829	4.91855
H	0.23737	3.61963	3.83723
H	2.00212	3.43608	3.81993
C	5.97184	1.49291	6.71872
H	6.48048	1.28977	5.78186
C	-3.88205	1.12195	5.78399
H	-4.40023	2.01325	5.44374
C	-3.79092	1.15907	9.36415
H	-4.29879	2.08091	9.09994
C	-4.57438	-0.04774	6.08843
H	-5.65627	-0.09111	5.99117
C	-4.49711	0.04402	9.80985
H	-5.57905	0.07861	9.90971
C	-2.41332	-1.12421	9.96773
H	-1.79861	-1.98935	10.18731

C	-3.79505	-1.11788	10.11978
H	-4.30377	-2.01151	10.46855
C	-2.47624	-1.06702	6.63258
H	-1.84515	-1.87493	6.98942
C	-3.85850	-1.16073	6.52295
H	-4.35713	-2.08953	6.78155
Co	1.94368	-1.41368	7.77728
N	3.88347	-1.61519	7.69225
O	1.84062	-2.76629	9.18450
O	1.70259	-2.64509	6.27497
O	0.11075	-1.22192	7.84261
O	0.21777	-1.33303	5.13986
O	0.39691	-1.59857	10.51693
C	1.14223	-2.58365	10.22359
C	0.93067	-2.36393	5.30683
C	6.64003	-1.81668	7.61033
H	7.72406	-1.89025	7.57801
C	5.99063	-1.45715	8.78906
H	6.54484	-1.23406	9.69484
C	4.49634	-1.95608	6.55428
H	3.83680	-2.13680	5.71408
C	4.60367	-1.36927	8.79097
H	4.02037	-1.06323	9.65324
C	0.89074	-3.39474	4.19455
H	0.89853	-4.40332	4.61699
H	0.01499	-3.24892	3.55870
H	1.79235	-3.28330	3.58019
C	1.23429	-3.67775	11.26998
H	2.15651	-3.53124	11.84457
H	0.38674	-3.63378	11.95775
H	1.29286	-4.65709	10.78766
C	5.87868	-2.07158	6.47230
H	6.34174	-2.34987	5.53052

--

-1 1

O	1.80963	2.61877	9.44937
O	0.36158	1.32442	10.65095
C	1.06936	2.35178	10.44603
C	1.05422	3.40805	11.53403
H	2.03830	3.43897	12.01559
H	0.29294	3.18614	12.28439
H	0.87696	4.39107	11.08728

Final Energy: -2788.0498404643

EDA for $[Co_4O_4(py)_4]^{+4}$ and $(OAc)_4$ bonds

```
-------------------------------------------------------------------
*                 Energy decomposition analysis                   *
*  R.Z.Khaliullin, E.A.Cobar, R.C.Lochan, A.T.Bell, M.Head-Gordon *
*            J. Phys. Chem. A, 2007, 111, 8753-8765.              *
-------------------------------------------------------------------
  Fragment          E(TOTAL)      E(RS CP-CORR)     E(SCF CP-CORR)
-------------------------------------------------------------------
         1   -2559.3899920496              N/A                N/A
         2    -228.4081907253              N/A                N/A
-------------------------------------------------------------------
  initial    -2787.8935152403
   SCF MI    -2787.9620129475
       RS    -2788.0504685445
      SCF    -2788.0498404643
-------------------------------------------------------------------
                                      Energy term         DE, kJ/mol
-------------------------------------------------------------------
                          Frozen Density ( FRZ )          -250.29836
                            Polarization ( POL )          -179.84286
                     RS Delocalization ( P-DEL )          -232.24293
                    SCF Delocalization ( V-DEL )          -230.59388
-------------------------------------------------------------------
          RS Total ( P-TOT = FRZ + POL + P-DEL )          -662.38415
         SCF Total ( V-TOT = FRZ + POL + V-DEL )          -660.73510
   Higher order relaxation ( HO = V-TOT - P-TOT )            1.64904

*-----------------------------------------------------------------*
*      Energy decomposition of the delocalization term, kJ/mol    *
*-----------------------------------------------------------------*
              DEL from fragment(row) to fragment(col)
 ------------------------------------------------------------------
           1           2
  1    -0.00003   -16.26554
  2  -215.97740    -0.00002
-------------------------------------------------------------------


-------------------------------------------------------------------
*                  Charge transfer analysis                       *
*           R.Z.Khaliullin, A.T. Bell, M.Head-Gordon              *
*              J. Chem. Phys., 2008, 128, 184112                  *
*-----------------------------------------------------------------*
                     Fragment population, a.u.
-------------------------------------------------------------------
   Method   Fragment          Occ          Vir     Occ+Vir    Isolated
-------------------------------------------------------------------
   SCF MI          1  360.000000    0.000000  360.000000  360.000000
                   2   32.000000    0.000000   32.000000   32.000000
-------------------------------------------------------------------
       RS          1  359.992424    0.262715  360.255140  360.000000
                   2   31.752399   -0.007539   31.744860   32.000000
-------------------------------------------------------------------
      SCF          1  359.984320    0.218400  360.202720  360.000000
```

```
                 2   31.795001    0.002279   31.797280   32.000000
--------------------------------------------------------------------
                         Total electron transfer            DQ, me-
--------------------------------------------------------------------
                               RS Delocalization         255.176627
                              SCF Delocalization         220.678569
            Higher order relaxation ( SCF - RS )         -34.498058


*------------------------------------------------------------------*
*      Decomposition of the total RS charge-transfer term, me-      *
*------------------------------------------------------------------*
         Delocalization from fragment(row) to fragment(col)
 ------------------------------------------------------------------
           1           2
  1     1.34553     6.22998
  2   261.36988   -13.76876
 ------------------------------------------------------------------
```

EDA for $[Co_4O_4(py)_4]^{+5}$ and $(OAc)_4$ bonds

```
--------------------------------------------------------------------
*                  Energy decomposition analysis                   *
*  R.Z.Khaliullin, E.A.Cobar, R.C.Lochan, A.T.Bell, M.Head-Gordon   *
*            J. Phys. Chem. A, 2007, 111, 8753-8765.               *
--------------------------------------------------------------------
  Fragment           E(TOTAL)      E(RS CP-CORR)     E(SCF CP-CORR)
--------------------------------------------------------------------
         1   -2559.0180870657               N/A                 N/A
         2    -228.4076309901               N/A                 N/A
--------------------------------------------------------------------
  initial    -2787.6178004145
   SCF MI    -2787.7028161378
       RS    -2787.8122199085
      SCF    -2787.8089877182
--------------------------------------------------------------------
                                     Energy term         DE, kJ/mol
--------------------------------------------------------------------
                          Frozen Density ( FRZ )         -504.31822
                            Polarization ( POL )         -223.21143
                     RS Delocalization ( P-DEL )         -287.24301
                    SCF Delocalization ( V-DEL )         -278.75679
--------------------------------------------------------------------
          RS Total ( P-TOT = FRZ + POL + P-DEL )        -1014.77266
         SCF Total ( V-TOT = FRZ + POL + V-DEL )        -1006.28644
  Higher order relaxation ( HO = V-TOT - P-TOT )            8.48622

*------------------------------------------------------------------*
*      Energy decomposition of the delocalization term, kJ/mol      *
*------------------------------------------------------------------*
              DEL from fragment(row) to fragment(col)
 ------------------------------------------------------------------
           1           2
  1    -0.00059   -18.41226
  2  -268.82835    -0.00030
--------------------------------------------------------------------
```

```
-------------------------------------------------------------------
*                  Charge transfer analysis                       *
*           R.Z.Khaliullin, A.T. Bell, M.Head-Gordon              *
*              J. Chem. Phys., 2008, 128, 184112                  *
*-----------------------------------------------------------------*
                    Fragment population, a.u.
-------------------------------------------------------------------
   Method   Fragment          Occ          Vir     Occ+Vir    Isolated
-------------------------------------------------------------------
   SCF MI          1  359.000000    0.000000  359.000000  359.000000
                   2   32.000000    0.000000   32.000000   32.000000
-------------------------------------------------------------------
       RS          1  358.983996    0.438306  359.422302  359.000000
                   2   31.586346   -0.008648   31.577698   32.000000
-------------------------------------------------------------------
      SCF          1  358.869092    0.422241  359.291333  359.000000
                   2   31.704973    0.003693   31.708667   32.000000
-------------------------------------------------------------------
                        Total electron transfer           DQ, me-
-------------------------------------------------------------------
                              RS Delocalization         429.657839
                             SCF Delocalization         425.934465
             Higher order relaxation ( SCF - RS )         -3.723374


*-----------------------------------------------------------------*
*      Decomposition of the total RS charge-transfer term, me-    *
*-----------------------------------------------------------------*
          Delocalization from fragment(row) to fragment(col)
 ------------------------------------------------------------------
            1           2
   1     9.73190     6.27207
   2   428.57393   -14.92006
 ------------------------------------------------------------------
```

EDA for $[Co_4O_4(OAc)_4]^0$ and $(py)_4$ bonds

```
-------------------------------------------------------------------
*                  Energy decomposition analysis                  *
*  R.Z.Khaliullin, E.A.Cobar, R.C.Lochan, A.T.Bell, M.Head-Gordon  *
*              J. Phys. Chem. A, 2007, 111, 8753-8765.            *
-------------------------------------------------------------------
  Fragment           E(TOTAL)      E(RS CP-CORR)     E(SCF CP-CORR)
-------------------------------------------------------------------
         1   -2539.7923503050               N/A               N/A
         2    -248.1878155028               N/A               N/A
-------------------------------------------------------------------
  initial    -2787.9805055210
   SCF MI    -2787.9976109372
       RS    -2788.0453851816
      SCF    -2788.0498404640
-------------------------------------------------------------------
                                     Energy term         DE, kJ/mol
-------------------------------------------------------------------
```

```
                          Frozen Density ( FRZ )           -0.89193
                            Polarization ( POL )          -44.91080
                     RS Delocalization ( P-DEL )         -125.43277
                    SCF Delocalization ( V-DEL )         -137.13025
---------------------------------------------------------------------
            RS Total ( P-TOT = FRZ + POL + P-DEL )        -171.23550
           SCF Total ( V-TOT = FRZ + POL + V-DEL )        -182.93298
   Higher order relaxation ( HO = V-TOT - P-TOT )          -11.69748

*-------------------------------------------------------------------*
*      Energy decomposition of the delocalization term, kJ/mol      *
*-------------------------------------------------------------------*
               DEL from fragment(row) to fragment(col)
 ------------------------------------------------------------------
           1           2
   1    -0.00002   -27.99909
   2   -97.43360    -0.00000
---------------------------------------------------------------------


---------------------------------------------------------------------
*                      Charge transfer analysis                     *
*              R.Z.Khaliullin, A.T. Bell, M.Head-Gordon             *
*                 J. Chem. Phys., 2008, 128, 184112                 *
*-------------------------------------------------------------------*
                      Fragment population, a.u.
---------------------------------------------------------------------
   Method   Fragment          Occ          Vir     Occ+Vir    Isolated
---------------------------------------------------------------------
   SCF MI          1  350.000000    0.000000  350.000000  350.000000
                   2   42.000000    0.000000   42.000000   42.000000
---------------------------------------------------------------------
       RS          1  349.980729    0.087656  350.068385  350.000000
                   2   41.916319    0.015296   41.931615   42.000000
---------------------------------------------------------------------
      SCF          1  349.969545    0.103513  350.073059  350.000000
                   2   41.900820    0.026122   41.926941   42.000000
---------------------------------------------------------------------
                          Total electron transfer           DQ, me-
---------------------------------------------------------------------
                                RS Delocalization        102.951355
                               SCF Delocalization        129.634897
             Higher order relaxation ( SCF - RS )         26.683541


*-------------------------------------------------------------------*
*      Decomposition of the total RS charge-transfer term, me-      *
*-------------------------------------------------------------------*
          Delocalization from fragment(row) to fragment(col)
 ------------------------------------------------------------------
           1           2
   1    -0.26356    19.53415
   2    87.91915    -4.23838
 ------------------------------------------------------------------
```

EDA for $[Co_4O_4(OAc)_4]^+$ and $(py)_4$ bonds

```
--------------------------------------------------------------------
 *                   Energy decomposition analysis                   *
 *  R.Z.Khaliullin, E.A.Cobar, R.C.Lochan, A.T.Bell, M.Head-Gordon   *
 *            J. Phys. Chem. A, 2007, 111, 8753-8765.                *
 --------------------------------------------------------------------
   Fragment           E(TOTAL)      E(RS CP-CORR)     E(SCF CP-CORR)
 --------------------------------------------------------------------
         1   -2539.5459129884               N/A                N/A
         2    -248.1877321153               N/A                N/A
 --------------------------------------------------------------------
   initial    -2787.7399711849
    SCF MI    -2787.7588007517
        RS    -2787.8065139521
       SCF    -2787.8105692569
 --------------------------------------------------------------------
                                        Energy term         DE, kJ/mol
 --------------------------------------------------------------------
                              Frozen Density ( FRZ )          -16.60932
                                Polarization ( POL )          -49.43761
                         RS Delocalization ( P-DEL )         -125.27249
                        SCF Delocalization ( V-DEL )         -135.91982
 --------------------------------------------------------------------
              RS Total ( P-TOT = FRZ + POL + P-DEL )         -191.31943
             SCF Total ( V-TOT = FRZ + POL + V-DEL )         -201.96676
   Higher order relaxation ( HO = V-TOT - P-TOT )             -10.64733

 *-------------------------------------------------------------------*
 *      Energy decomposition of the delocalization term, kJ/mol      *
 *-------------------------------------------------------------------*
               DEL from fragment(row) to fragment(col)
  ------------------------------------------------------------------
           1           2
   1    -0.00717   -23.35990
   2  -101.91759     0.00015
 --------------------------------------------------------------------


 --------------------------------------------------------------------
 *                   Charge transfer analysis                        *
 *           R.Z.Khaliullin, A.T. Bell, M.Head-Gordon                *
 *               J. Chem. Phys., 2008, 128, 184112                   *
 *-------------------------------------------------------------------*
                      Fragment population, a.u.
 --------------------------------------------------------------------
   Method   Fragment          Occ          Vir     Occ+Vir    Isolated
 --------------------------------------------------------------------
   SCF MI          1  349.000000    0.000000  349.000000  349.000000
                   2   42.000000    0.000000   42.000000   42.000000
 --------------------------------------------------------------------
       RS          1  348.986672    0.103437  349.090108  349.000000
                   2   41.901711    0.008181   41.909892   42.000000
 --------------------------------------------------------------------
      SCF          1  348.978267    0.118069  349.096336  349.000000
```

```
                2   41.887951    0.015713   41.903664   42.000000
---------------------------------------------------------------------
                         Total electron transfer            DQ, me-
---------------------------------------------------------------------
                               RS Delocalization         111.617805
                              SCF Delocalization         133.782042
             Higher order relaxation ( SCF - RS )         22.164237


*-------------------------------------------------------------------*
*      Decomposition of the total RS charge-transfer term, me-      *
*-------------------------------------------------------------------*
          Delocalization from fragment(row) to fragment(col)
  ------------------------------------------------------------------
              1           2
    1    -0.16639    13.49470
    2   103.60315    -5.31365
  ------------------------------------------------------------------
```

Coordinates and energy for 4OAc-4CNpy

```
0 1
--
0 1
Co         0.21468       -0.07646        9.34227
Co         0.13961        0.06233        6.54328
Co         2.00000        1.35273        7.96996
O          1.97995        0.01239        6.67284
O          2.04496       -0.10667        9.11109
N          3.95152        1.53414        7.96992
O          1.86477        2.70151        6.56365
O          0.16431        1.21005        7.99620
O          0.31765        1.57132        5.31816
N         -1.72164       -0.02458        9.57365
N         -1.81024        0.07460        6.37464
C         -2.47758        1.15166        5.94758
H         -1.86458        2.01832        5.73075
C          1.11082        2.53431        5.56072
C          6.69768        1.86151        7.89717
C         -2.37566        1.10204        9.26820
H         -1.74388        1.91621        8.92896
C          5.99426        2.03317        9.09585
H          6.50861        2.30218       10.01180
C          4.61587        1.35835        6.82365
H          3.99753        1.06999        5.98000
C          4.61795        1.85576        9.08532
H          4.00425        1.97904        9.96895
C          1.18018        3.59899        4.48555
H          1.59635        4.52724        4.88155
H          0.18920        3.77141        4.05702
H          1.82770        3.23416        3.67947
C          5.99288        1.50892        6.74018
H          6.50784        1.35538        5.79861
C         -3.85573        1.14313        5.77987
H         -4.37446        2.02777        5.42744
C         -3.76020        1.18068        9.32119
```

```
H          -4.27168        2.09586        9.04572
C          -4.55657       -0.03388        6.07088
C          -4.47965        0.04589        9.71641
C          -2.39718       -1.10640        9.97164
H          -1.78723       -1.96932       10.21084
C          -3.78158       -1.11754       10.06010
H          -4.30788       -2.01211       10.37463
C          -2.47025       -1.05506        6.65618
H          -1.84723       -1.86608        7.01966
C          -3.84752       -1.15361        6.52241
H          -4.36026       -2.07829        6.76298
Co          1.92800       -1.44603        7.81881
O           1.82111       -2.78336        9.23239
O           1.66090       -2.67730        6.32618
O           0.10139       -1.22473        7.89081
O           0.19540       -1.34555        5.19104
O           0.39329       -1.59325       10.56138
C           1.12910       -2.58916       10.27437
C           0.88810       -2.39117        5.35922
C           0.82195       -3.42395        4.25275
H           0.89494       -4.43140        4.66999
H          -0.09475       -3.31071        3.67038
H           1.67669       -3.27340        3.58224
C           1.20999       -3.66685       11.33559
H           1.95274       -3.36203       12.08224
H           0.25020       -3.77233       11.84798
H           1.52179       -4.61769       10.89868
O           1.85961        2.59328        9.47045
O           0.39484        1.32623       10.67974
C           1.10633        2.35060       10.46413
C           1.02358        3.44415       11.50792
H           1.94605        4.02897       11.53005
H           0.80526        3.02152       12.49111
H           0.20306        4.11774       11.23306
C          -5.91083        0.06232        9.74884
C          -5.97762       -0.09847        5.90757
C           8.11633        2.04210        7.85242
N          -7.07318        0.06405        9.76927
N          -7.13023       -0.15849        5.76907
N           9.26771        2.19675        7.81505
--
0 1
N           3.85806       -1.65644        7.69689
C           6.60981       -1.74775        7.49587
C           5.99742       -1.46959        8.72394
H           6.58942       -1.26720        9.60901
C           4.43281       -1.93204        6.52144
H           3.74761       -2.09773        5.69976
C           4.61095       -1.43694        8.77876
C           5.80978       -1.99256        6.37363
H           4.05503       -1.18915        9.67651
H           6.25237       -2.21038        5.40794
C           8.03530       -1.75438        7.37512
N           9.19226       -1.75091        7.26263
```

Final Energy: -3156.8392564770

Coordinates and energy for $[4OAc\text{-}4CNpy]^{+}$

1 2
--
1 2

C	-1.21280	-1.62528	2.83119
C	-1.66390	-2.31731	4.09150
H	-1.33759	-1.72322	4.95250
H	-1.18842	-3.29859	4.17002
H	-2.75095	-2.40861	4.11293
C	1.19784	1.72258	-2.73487
C	1.79007	2.43519	-3.92422
H	2.80825	2.06562	-4.08681
H	1.80884	3.51366	-3.76233
H	1.21295	2.19732	-4.82275
C	2.11326	-2.62350	-0.71388
C	3.08221	-3.74632	-0.98172
H	4.07789	-3.31192	-1.13236
H	2.80419	-4.27584	-1.89513
H	3.12843	-4.42986	-0.13162
C	1.96527	0.54230	3.35517
H	0.90583	0.74379	3.46403
C	2.91780	1.04785	4.22646
H	2.62029	1.65888	5.07106
C	4.26606	0.75482	3.98733
C	4.60372	-0.03607	2.88273
H	5.63682	-0.28639	2.66891
C	3.58527	-0.49537	2.06071
H	3.77499	-1.11321	1.19251
C	-3.65110	-2.05295	-0.11342
H	-3.21865	-2.51340	0.76572
C	-4.68540	-2.63992	-0.82617
H	-5.09445	-3.59542	-0.51718
C	-5.17565	-1.97820	-1.95834
C	-4.62931	-0.73798	-2.31225
H	-4.99229	-0.19666	-3.17860
C	-3.60152	-0.22067	-1.53963
H	-3.10523	0.71443	-1.77035
C	-1.37050	-2.72850	-2.44502
H	-1.02099	-3.14414	-1.50701
C	-2.23708	-3.40960	-3.28611
H	-2.58517	-4.40365	-3.02904
C	-2.64698	-2.78856	-4.47264
C	-2.17274	-1.50396	-4.76412
H	-2.46616	-0.99425	-5.67516
C	-1.31478	-0.89256	-3.86212
H	-0.90817	0.09648	-4.02769
N	2.30029	-0.20739	2.29788
N	-3.11989	-0.88105	-0.47908
N	-0.92757	-1.49823	-2.73266
O	-0.45955	-1.48490	0.01095
O	-0.19805	0.63189	1.39935
O	1.46489	0.17715	-0.28861
O	-0.94037	0.67306	-1.05171
O	0.04789	-1.68228	2.60755
O	-2.09292	-1.05036	2.13250

```
O           0.95010        2.42752       -1.71165
O           1.01872        0.46844       -2.88097
O           1.55324       -2.09173       -1.70883
O           1.98220       -2.29756        0.52057
Co          0.84912       -0.84622        1.08003
Co         -1.63778       -0.11220        0.48022
Co          0.29652        1.64546       -0.09164
Co          0.28173       -0.63370       -1.49784
O          -2.68476        1.44853        0.82399
O          -1.06690        2.95074        0.24804
C          -2.23743        2.61934        0.61942
C          -3.21129        3.74286        0.86876
H          -3.44753        3.77642        1.93789
H          -4.14612        3.53972        0.33837
H          -2.79561        4.69983        0.55058
C           5.28215        1.26895        4.85199
C          -3.54210       -3.45510       -5.36834
C          -6.20126       -2.57319       -2.75976
N           6.10772        1.69333        5.55095
N          -4.27091       -3.99547       -6.09457
N          -7.01708       -3.06671       -3.42431
--
0 1
C           1.22972        3.46976        1.94038
H           0.16097        3.55616        2.09062
C           2.14525        4.15302        2.72606
H           1.80774        4.80040        3.52785
C           3.50903        3.97975        2.46343
C           3.89806        3.14317        1.40992
H           4.94554        2.99127        1.17589
C           2.91234        2.50546        0.67268
H           3.13957        1.82736       -0.14172
N           5.28528        5.14026        3.95212
C           4.49064        4.62837        3.27614
N           1.61073        2.66040        0.94469

Final Energy: -3156.5708294335
```

EDA for $[Co_4O_4(OAc)_4]^0$ and $(CNpy)_4$ bonds

```
 --------------------------------------------------------------------
 *                   Energy decomposition analysis                  *
 *  R.Z.Khaliullin, E.A.Cobar, R.C.Lochan, A.T.Bell, M.Head-Gordon   *
 *            J. Phys. Chem. A, 2007, 111, 8753-8765.               *
 --------------------------------------------------------------------
  Fragment            E(TOTAL)      E(RS CP-CORR)     E(SCF CP-CORR)
 --------------------------------------------------------------------
         1   -2816.3823847534               N/A                N/A
         2    -340.3863226046               N/A                N/A
 --------------------------------------------------------------------
  initial    -3156.7676608236
   SCF MI    -3156.7847534908
       RS    -3156.8344063997
      SCF    -3156.8392564770
 --------------------------------------------------------------------
                                      Energy term         DE, kJ/mol
```

```
-------------------------------------------------------------------
                          Frozen Density ( FRZ )            2.74771
                            Polarization ( POL )          -44.87733
                     RS Delocalization ( P-DEL )         -130.36526
                    SCF Delocalization ( V-DEL )         -143.09929
-------------------------------------------------------------------
          RS Total ( P-TOT = FRZ + POL + P-DEL )         -172.49488
         SCF Total ( V-TOT = FRZ + POL + V-DEL )         -185.22891
   Higher order relaxation ( HO = V-TOT - P-TOT )         -12.73403

*-----------------------------------------------------------------*
*      Energy decomposition of the delocalization term, kJ/mol     *
*-----------------------------------------------------------------*
               DEL from fragment(row) to fragment(col)
 -----------------------------------------------------------------
            1           2
   1    -0.00002   -31.20637
   2   -99.15883    -0.00000
-------------------------------------------------------------------


-------------------------------------------------------------------
*                   Charge transfer analysis                       *
*            R.Z.Khaliullin, A.T. Bell, M.Head-Gordon              *
*               J. Chem. Phys., 2008, 128, 184112                  *
*-----------------------------------------------------------------*
                        Fragment population, a.u.
-------------------------------------------------------------------
   Method   Fragment          Occ          Vir     Occ+Vir    Isolated
-------------------------------------------------------------------
   SCF MI          1  386.000000    0.000000  386.000000  386.000000
                   2   54.000000    0.000000   54.000000   54.000000
-------------------------------------------------------------------
       RS          1  385.976407    0.085674  386.062081  386.000000
                   2   53.917937    0.019982   53.937919   54.000000
-------------------------------------------------------------------
      SCF          1  385.961959    0.102934  386.064893  386.000000
                   2   53.900967    0.034140   53.935107   54.000000
-------------------------------------------------------------------
                           Total electron transfer           DQ, me-
-------------------------------------------------------------------
                                 RS Delocalization        105.656232
                                SCF Delocalization        137.073858
             Higher order relaxation ( SCF - RS )          31.417627


*-----------------------------------------------------------------*
*      Decomposition of the total RS charge-transfer term, me-     *
*-----------------------------------------------------------------*
          Delocalization from fragment(row) to fragment(col)
 -----------------------------------------------------------------
            1           2
   1    -0.32733    23.92066
   2    86.00158    -3.93868
 -----------------------------------------------------------------
```

EDA for $[Co_4O_4(OAc)_4]^+$ and $(CNpy)_4$ bonds

```
-------------------------------------------------------------------
 *                   Energy decomposition analysis                  *
 *  R.Z.Khaliullin, E.A.Cobar, R.C.Lochan, A.T.Bell, M.Head-Gordon   *
 *             J. Phys. Chem. A, 2007, 111, 8753-8765.              *
 -------------------------------------------------------------------
   Fragment           E(TOTAL)      E(RS CP-CORR)     E(SCF CP-CORR)
 -------------------------------------------------------------------
          1   -2816.1018911484               N/A                N/A
          2    -340.3866107261               N/A                N/A
 -------------------------------------------------------------------
   initial    -3156.4905522801
    SCF MI    -3156.5131364760
        RS    -3156.5660134943
       SCF    -3156.5708294335
 -------------------------------------------------------------------
                                       Energy term         DE, kJ/mol
 -------------------------------------------------------------------
                           Frozen Density ( FRZ )           -5.38340
                             Polarization ( POL )          -59.29551
                      RS Delocalization ( P-DEL )         -138.83026
                     SCF Delocalization ( V-DEL )         -151.47466
 -------------------------------------------------------------------
           RS Total ( P-TOT = FRZ + POL + P-DEL )         -203.50917
          SCF Total ( V-TOT = FRZ + POL + V-DEL )         -216.15357
    Higher order relaxation ( HO = V-TOT - P-TOT )          -12.64440

 *-----------------------------------------------------------------*
 *      Energy decomposition of the delocalization term, kJ/mol     *
 *-----------------------------------------------------------------*
              DEL from fragment(row) to fragment(col)
  ------------------------------------------------------------------
              1           2
   1    -0.00003   -25.10605
   2  -113.72476     0.00004
 -------------------------------------------------------------------

-------------------------------------------------------------------
 *                     Charge transfer analysis                     *
 *           R.Z.Khaliullin, A.T. Bell, M.Head-Gordon               *
 *               J. Chem. Phys., 2008, 128, 184112                  *
 *-----------------------------------------------------------------*
                       Fragment population, a.u.
 -------------------------------------------------------------------
    Method   Fragment          Occ          Vir     Occ+Vir    Isolated
 -------------------------------------------------------------------
    SCF MI          1  385.000000    0.000000  385.000000  385.000000
                    2   54.000000    0.000000   54.000000   54.000000
 -------------------------------------------------------------------
        RS          1  384.984848    0.114783  385.099631  385.000000
                    2   53.890171    0.010198   53.900369   54.000000
 -------------------------------------------------------------------
       SCF          1  384.967882    0.139234  385.107116  385.000000
                    2   53.873093    0.019791   53.892884   54.000000
 -------------------------------------------------------------------
```

```
                             Total electron transfer           DQ, me-
 ---------------------------------------------------------------------
                                   RS Delocalization        124.981186
                                  SCF Delocalization        159.024812
               Higher order relaxation ( SCF - RS )          34.043626


 *--------------------------------------------------------------------*
 *      Decomposition of the total RS charge-transfer term, me-      *
 *--------------------------------------------------------------------*
           Delocalization from fragment(row) to fragment(col)
  -------------------------------------------------------------------
             1           2
    1    -0.14270    15.29500
    2   114.92561    -5.09673
  -------------------------------------------------------------------
```

Coordinates and energy for 4TFA-4py

```
0 1
--
1 1
Co         0.20123      -0.05729       9.28515
Co         0.14015       0.03847       6.49682
Co         1.99300       1.37825       7.90421
O          1.98814       0.02178       6.63356
O          2.04002      -0.05742       9.06576
N          3.93958       1.59138       7.89669
O          1.80616       2.73819       6.49483
O          0.15199       1.20579       7.92652
O          0.30261       1.53601       5.25136
N         -1.73289      -0.02880       9.53310
N         -1.80583       0.03473       6.33128
C         -2.47746       1.10510       5.88980
H         -1.87328       1.96703       5.63333
C          1.01902       2.52868       5.54371
C          6.68845       1.90570       7.80754
H          7.76723       2.03146       7.77127
C         -2.39754       1.09238       9.22719
H         -1.78315       1.89462       8.83278
C          5.98083       2.19157       8.97246
H          6.48094       2.54670       9.86780
C          4.61109       1.31234       6.77368
H          3.99963       0.94523       5.95580
C          4.60181       2.02090       8.97891
H          3.99153       2.24116       9.84617
C          0.83787       3.68975       4.54167
C          5.99024       1.45559       6.68955
H          6.49842       1.21254       5.76238
C         -3.86082       1.09265       5.75565
H         -4.37266       1.97757       5.39132
C         -3.77659       1.18309       9.36648
H         -4.28447       2.10255       9.09533
C         -4.55952      -0.06430       6.09178
H         -5.64144      -0.10365       5.99710
C         -4.47822       0.07907       9.84567
H         -5.55779       0.11958       9.96365
C         -2.39571      -1.09344       9.99877
```

H	-1.78861	-1.95859	10.23729
C	-3.77465	-1.07779	10.16992
H	-4.27846	-1.96134	10.54892
C	-2.46747	-1.08330	6.65464
H	-1.84581	-1.89098	7.02663
C	-3.84979	-1.17131	6.55079
H	-4.35189	-2.09197	6.82965
Co	1.97302	-1.41342	7.79741
N	3.90911	-1.62047	7.72055
O	1.84096	-2.76521	9.21666
O	1.73735	-2.66698	6.31413
O	0.13352	-1.22600	7.85580
O	0.25501	-1.37654	5.13837
O	0.40183	-1.55664	10.52759
C	1.09513	-2.55469	10.20033
C	1.00698	-2.35776	5.33739
C	6.66508	-1.83432	7.66063
H	7.74858	-1.91391	7.63657
C	6.01230	-1.42042	8.81946
H	6.56179	-1.16233	9.71841
C	4.52532	-2.02039	6.60157
H	3.87838	-2.25108	5.76395
C	4.62648	-1.32807	8.81113
H	4.04540	-0.98487	9.66037
C	1.11957	-3.36784	4.17401
C	0.93755	-3.73160	11.18804
C	5.90763	-2.14098	6.53303
H	6.37189	-2.46837	5.60826
F	-0.44103	4.12097	4.60691
F	1.63855	4.72505	4.79834
F	1.07048	3.27572	3.28766
F	0.25537	-3.11922	3.18864
F	0.91948	-4.62092	4.60521
F	2.36942	-3.29719	3.66457
F	1.74729	-4.75360	10.90698
F	1.17559	-3.33706	12.44675
F	-0.33729	-4.17616	11.12492

--

-1 1

O	1.82348	2.63415	9.39429
O	0.38489	1.35384	10.63411
C	1.12635	2.33474	10.39798
C	1.26342	3.36619	11.53823
F	2.54205	3.36317	11.97343
F	0.47054	3.09578	12.57632
F	0.97806	4.60026	11.09856

Final Energy: -3978.4703578200

Coordinates and energy for [4TFA-4py]$^+$

```
1 2
--
2 2
C         -1.20568       -1.63511        2.77711
C         -1.77828       -2.50872        3.91812
C          1.15975        1.68316       -2.74146
C          1.54977        2.39398       -4.05722
C          2.16224       -2.57519       -0.68708
C          3.26036       -3.63905       -0.91899
C          1.96161        0.57349        3.33887
H          0.90886        0.82123        3.40922
C          2.91111        1.05570        4.22813
H          2.59969        1.69491        5.04711
C          4.24587        0.70027        4.04472
H          5.00996        1.05699        4.72924
C          4.58490       -0.12282        2.97344
H          5.60996       -0.42963        2.79445
C          3.58210       -0.56584        2.12133
H          3.78148       -1.21998        1.28237
C          1.27180        3.53298        1.86610
H          0.20396        3.64700        2.00673
C          2.19766        4.25581        2.60673
H          1.85096        4.95526        3.36029
C          3.55446        4.06845        2.35475
H          4.30128        4.62282        2.91571
C          3.93852        3.16408        1.36665
H          4.98308        2.98851        1.13305
C          2.95477        2.48140        0.66492
H          3.18351        1.75575       -0.10731
C         -3.67878       -2.02220       -0.05624
H         -3.25269       -2.47779        0.82873
C         -4.77083       -2.56656       -0.71871
H         -5.21730       -3.48432       -0.35062
C         -5.27146       -1.91264       -1.84192
H         -6.12615       -2.31483       -2.37838
C         -4.66387       -0.73199       -2.26430
H         -5.02231       -0.18925       -3.13240
C         -3.57852       -0.24383       -1.55057
H         -3.04463        0.65683       -1.82974
C         -1.37926       -2.73738       -2.39705
H         -1.05669       -3.13396       -1.44123
C         -2.24977       -3.41932       -3.23593
H         -2.62006       -4.39611       -2.94390
C         -2.62158       -2.83018       -4.44257
H         -3.29382       -3.34464       -5.12332
C         -2.11278       -1.57466       -4.76496
H         -2.36933       -1.08039       -5.69611
C         -1.25141       -0.94815       -3.87395
H         -0.81442        0.02085       -4.07724
N          2.30189       -0.21759        2.31161
N          1.65167        2.66371        0.91918
N         -3.10230       -0.88835       -0.47670
N         -0.89930       -1.52816       -2.71769
O         -0.43308       -1.48404        0.02899
O         -0.17523        0.64858        1.38710
```

```
O          1.49959        0.16946       -0.28442
O         -0.90130        0.65544       -1.06067
O          0.04417       -1.70257        2.61265
O         -2.08500       -1.01863        2.13030
O          0.96980        2.41877       -1.74525
O          1.04841        0.43328       -2.88066
O          1.61127       -2.10137       -1.70221
O          1.99725       -2.30895        0.54053
Co         0.87379       -0.82152        1.08525
Co        -1.61888       -0.11371        0.46472
Co         0.34356        1.63967       -0.09840
Co         0.30523       -0.66259       -1.49560
F          0.52372        2.24993       -4.91943
F          1.77897        3.68994       -3.87733
F          2.63498        1.82642       -4.58897
F          4.43152       -3.12122       -0.49958
F          3.37068       -3.96749       -2.20046
F          2.99836       -4.73619       -0.20479
F         -2.44943       -3.52572        3.34348
F         -0.82479       -3.00865        4.69671
F         -2.62911       -1.80219        4.66349
--
-1 1
O         -2.66035        1.46203        0.81046
O         -1.02647        2.94987        0.21766
C         -2.16757        2.60143        0.62221
C         -3.07331        3.79781        0.99063
F         -3.10835        4.68008       -0.01041
F         -2.54236        4.39586        2.07434
F         -4.31367        3.41490        1.27678

Final Energy: -3978.1925394789
```

EDA for $[Co_4O_4(py_4]^{+4}$ and $(TFA)_4$ bonds

```
 -------------------------------------------------------------------
 *                  Energy decomposition analysis                  *
 *  R.Z.Khaliullin, E.A.Cobar, R.C.Lochan, A.T.Bell, M.Head-Gordon  *
 *             J. Phys. Chem. A, 2007, 111, 8753-8765.             *
 -------------------------------------------------------------------
   Fragment           E(TOTAL)      E(RS CP-CORR)     E(SCF CP-CORR)
 -------------------------------------------------------------------
          1   -3452.1817388443               N/A                N/A
          2    -526.0371671990               N/A                N/A
 -------------------------------------------------------------------
   initial    -3978.3238717465
    SCF MI    -3978.3849096703
        RS    -3978.4698500123
       SCF    -3978.4703578200
 -------------------------------------------------------------------
                                       Energy term         DE, kJ/mol
 -------------------------------------------------------------------
                              Frozen Density ( FRZ )        -275.59072
                                Polarization ( POL )        -160.25697
                         RS Delocalization ( P-DEL )        -223.01351
```

```
                  SCF Delocalization ( V-DEL )         -224.34678
--------------------------------------------------------------------
           RS Total  ( P-TOT = FRZ + POL + P-DEL )       -658.86121
          SCF Total  ( V-TOT = FRZ + POL + V-DEL )       -660.19447
   Higher order relaxation ( HO = V-TOT - P-TOT )          -1.33327

*------------------------------------------------------------------*
*      Energy decomposition of the delocalization term, kJ/mol      *
*------------------------------------------------------------------*
               DEL from fragment(row) to fragment(col)
 ------------------------------------------------------------------
            1           2
   1    -0.00003   -17.24215
   2  -205.77136    -0.00001
--------------------------------------------------------------------


--------------------------------------------------------------------
*                    Charge transfer analysis                       *
*            R.Z.Khaliullin, A.T. Bell, M.Head-Gordon               *
*               J. Chem. Phys., 2008, 128, 184112                   *
*------------------------------------------------------------------*
                     Fragment population, a.u.
--------------------------------------------------------------------
   Method   Fragment          Occ          Vir      Occ+Vir    Isolated
--------------------------------------------------------------------
   SCF MI          1  432.000000    0.000000  432.000000  432.000000
                   2   56.000000    0.000000   56.000000   56.000000
--------------------------------------------------------------------
       RS          1  431.992205    0.228808  432.221013  432.000000
                   2   55.784747   -0.005759   55.778987   56.000000
--------------------------------------------------------------------
      SCF          1  431.984148    0.199630  432.183778  432.000000
                   2   55.813016    0.003206   55.816222   56.000000
--------------------------------------------------------------------
                          Total electron transfer           DQ, me-
--------------------------------------------------------------------
                                RS Delocalization        223.048441
                               SCF Delocalization        202.836490
           Higher order relaxation ( SCF - RS )          -20.211951


*------------------------------------------------------------------*
*      Decomposition of the total RS charge-transfer term, me-      *
*------------------------------------------------------------------*
          Delocalization from fragment(row) to fragment(col)
 ------------------------------------------------------------------
            1           2
   1     0.95321     6.84216
   2   227.85468   -12.60161
 ------------------------------------------------------------------
```

EDA for $[Co_4O_4(py_4]^{+5}$ and $(TFA)_4$ bonds

```
 -------------------------------------------------------------------
 *                  Energy decomposition analysis                   *
 *  R.Z.Khaliullin, E.A.Cobar, R.C.Lochan, A.T.Bell, M.Head-Gordon   *
 *             J. Phys. Chem. A, 2007, 111, 8753-8765.              *
 -------------------------------------------------------------------
   Fragment           E(TOTAL)      E(RS CP-CORR)     E(SCF CP-CORR)
 -------------------------------------------------------------------
          1   -3451.7821055383              N/A                 N/A
          2    -526.0354388444              N/A                 N/A
 -------------------------------------------------------------------
   initial    -3978.0135812992
    SCF MI    -3978.0905242287
        RS    -3978.1936848060
       SCF    -3978.1925394789
 -------------------------------------------------------------------
                                      Energy term          DE, kJ/mol
 -------------------------------------------------------------------
                           Frozen Density ( FRZ )          -514.70103
                             Polarization ( POL )          -202.01606
                      RS Delocalization ( P-DEL )          -270.85131
                     SCF Delocalization ( V-DEL )          -267.84422
 -------------------------------------------------------------------
            RS Total ( P-TOT = FRZ + POL + P-DEL )         -987.56840
           SCF Total ( V-TOT = FRZ + POL + V-DEL )         -984.56131
    Higher order relaxation ( HO = V-TOT - P-TOT )            3.00709

 *------------------------------------------------------------------*
 *      Energy decomposition of the delocalization term, kJ/mol      *
 *------------------------------------------------------------------*
                  DEL from fragment(row) to fragment(col)
  -----------------------------------------------------------------
               1           2
    1    -0.00128   -19.33429
    2  -251.51953    -0.00023
 -------------------------------------------------------------------


 -------------------------------------------------------------------
 *                    Charge transfer analysis                      *
 *             R.Z.Khaliullin, A.T. Bell, M.Head-Gordon             *
 *                J. Chem. Phys., 2008, 128, 184112                 *
 *------------------------------------------------------------------*
                      Fragment population, a.u.
 -------------------------------------------------------------------
    Method   Fragment          Occ          Vir     Occ+Vir    Isolated
 -------------------------------------------------------------------
    SCF MI          1  431.000000    0.000000  431.000000  431.000000
                    2   56.000000    0.000000   56.000000   56.000000
 -------------------------------------------------------------------
        RS          1  430.986002    0.365974  431.351975  431.000000
                    2   55.655204   -0.007179   55.648025   56.000000
 -------------------------------------------------------------------
       SCF          1  430.901333    0.361413  431.262746  431.000000
                    2   55.733899    0.003356   55.737254   56.000000
```

```
---------------------------------------------------------------------
                          Total electron transfer          DQ, me-
---------------------------------------------------------------------
                                 RS Delocalization        358.794648
                                SCF Delocalization        364.768571
             Higher order relaxation ( SCF - RS )           5.973923


*-------------------------------------------------------------------*
*      Decomposition of the total RS charge-transfer term, me-      *
*-------------------------------------------------------------------*
          Delocalization from fragment(row) to fragment(col)
 ------------------------------------------------------------------
             1            2
   1     7.27130      6.72718
   2   358.70225    -13.90608
 ------------------------------------------------------------------
```

Coordinates and energy for triplet [4OAc-4py]$^{2+}$

C	-1.39278	-1.54850	2.80858
C	-1.92755	-2.22901	4.03936
H	-1.64393	-1.63755	4.91686
H	-1.47538	-3.21740	4.14608
H	-3.01437	-2.29564	3.99337
C	1.31673	1.54794	-2.80791
C	1.92830	2.25179	-3.98884
H	3.01841	2.16692	-3.91087
H	1.65883	3.30829	-3.98987
H	1.62194	1.76353	-4.91525
C	-2.13239	2.72392	0.56346
C	-2.99552	3.92652	0.83267
H	-2.89953	4.18541	1.89333
H	-4.04227	3.68525	0.64102
H	-2.66878	4.77763	0.23471
C	2.09480	-2.69632	-0.54145
C	3.06501	-3.83029	-0.73138
H	4.08142	-3.42315	-0.68279
H	2.92199	-4.29080	-1.70919
H	2.95705	-4.56092	0.07199
C	1.96535	0.03422	3.67227
H	0.92916	-0.03232	3.97550
C	2.97361	0.40054	4.55620
H	2.72455	0.63481	5.58514
C	4.29103	0.44664	4.09657
H	5.09919	0.71929	4.76797
C	4.55282	0.13334	2.76105
H	5.56025	0.15732	2.36087
C	3.49504	-0.22660	1.93531
H	3.63462	-0.47693	0.89204
C	1.29722	3.20358	2.04256
H	0.31111	2.93400	2.39773
C	2.14854	4.02646	2.76871
H	1.82202	4.42691	3.72196
C	3.40651	4.32844	2.24263

H	4.08604	4.98136	2.78125
C	3.77481	3.78095	1.01206
H	4.74107	3.98879	0.56638
C	2.87245	2.96236	0.34285
H	3.09784	2.52043	-0.61801
C	-3.47302	-2.10576	-0.31597
H	-2.82075	-2.66587	0.34120
C	-4.57335	-2.68408	-0.93716
H	-4.80130	-3.72915	-0.75950
C	-5.36677	-1.89810	-1.77455
H	-6.23817	-2.32116	-2.26441
C	-5.02325	-0.55906	-1.96954
H	-5.61006	0.08724	-2.61263
C	-3.90839	-0.04869	-1.31533
H	-3.59651	0.98122	-1.42567
C	-1.05955	-2.92319	-2.65640
H	-0.57304	-3.43147	-1.83413
C	-1.83924	-3.59920	-3.58728
H	-1.96948	-4.67207	-3.49946
C	-2.43099	-2.87665	-4.62478
H	-3.03386	-3.38051	-5.37371
C	-2.23185	-1.49607	-4.68529
H	-2.67511	-0.89482	-5.47114
C	-1.43896	-0.88758	-3.71925
H	-1.24755	0.17745	-3.71707
N	2.22813	-0.27754	2.38921
N	1.65528	2.69210	0.84955
N	-3.15697	-0.81174	-0.49987
N	-0.85716	-1.59523	-2.73315
O	-0.49507	-1.44854	0.03430
O	-0.19062	0.69712	1.36975
O	1.48135	0.13740	-0.25340
O	-0.87163	0.65994	-1.08758
O	-0.12404	-1.67105	2.61423
O	-2.20758	-0.90603	2.08052
O	-2.63410	1.57954	0.78708
O	-0.93829	2.96722	0.14878
O	1.04487	2.28994	-1.79076
O	1.14080	0.29448	-2.89541
O	1.58586	-2.17231	-1.57902
O	1.88491	-2.33999	0.67849
Co	0.78606	-0.86113	1.15407
Co	-1.68088	-0.04503	0.45573
Co	0.34880	1.59752	-0.16079
Co	0.33178	-0.73193	-1.49500

Energy: -2790.04538213174

Spin Densities for triplet $[4OAc\text{-}4py]^{2+}$

Ground-State Mulliken Net Atomic Charges

Atom		Charge (a.u.)	Spin (a.u.)
1	C	0.877958	-0.005661
2	C	-0.789339	-0.003004
3	H	0.194166	0.000268
4	H	0.200756	0.000095
5	H	0.195470	-0.000683
6	C	0.875002	-0.004521
7	C	-0.803692	-0.002806
8	H	0.199320	0.000304
9	H	0.198159	0.000122
10	H	0.195592	-0.000599
11	C	0.791898	-0.006634
12	C	-0.850654	-0.002482
13	H	0.200166	0.000334
14	H	0.194756	-0.000606
15	H	0.198135	0.000139
16	C	0.798391	-0.007888
17	C	-0.857918	-0.002934
18	H	0.197206	0.000340
19	H	0.194937	-0.000668
20	H	0.200031	0.000116
21	C	0.289138	0.002485
22	H	0.166024	-0.000573
23	C	-0.151099	-0.004015
24	H	0.170525	-0.000065
25	C	-0.277957	0.008432
26	H	0.178525	-0.000464
27	C	-0.247504	0.001874
28	H	0.166140	-0.000037
29	C	0.288559	0.009885
30	H	0.164342	-0.000451
31	C	0.305994	0.009427
32	H	0.164421	-0.000465
33	C	-0.234985	0.002047
34	H	0.166559	-0.000027
35	C	-0.275803	0.009021
36	H	0.178713	-0.000497
37	C	-0.160755	-0.003285
38	H	0.170501	-0.000057
39	C	0.313691	0.001662
40	H	0.165401	-0.000603
41	C	0.267317	-0.004147
42	H	0.160609	-0.000152
43	C	-0.246960	-0.006918
44	H	0.163485	0.000054
45	C	-0.238701	-0.000660
46	H	0.174211	0.000032
47	C	-0.208883	-0.006996
48	H	0.166494	0.000063
49	C	0.278017	-0.002456
50	H	0.160323	-0.000102
51	C	0.289865	-0.002942

52	H	0.160901	-0.000126
53	C	-0.244359	-0.007707
54	H	0.165206	0.000060
55	C	-0.240649	-0.000584
56	H	0.174654	0.000032
57	C	-0.223458	-0.006769
58	H	0.165477	0.000054
59	C	0.268004	-0.003303
60	H	0.159440	-0.000114
61	N	0.172350	-0.024005
62	N	0.164206	-0.024574
63	N	0.136468	0.005670
64	N	0.139571	0.005756
65	O	-0.610812	0.017783
66	O	-0.578588	-0.086371
67	O	-0.578918	-0.085732
68	O	-0.589035	0.027912
69	O	-0.141498	-0.013571
70	O	-0.194830	0.009677
71	O	-0.163573	0.010978
72	O	-0.142803	-0.014927
73	O	-0.148001	-0.015603
74	O	-0.184465	0.009087
75	O	-0.166701	0.012030
76	O	-0.142006	-0.013413
77	Co	-0.121941	1.045018
78	Co	-0.018094	0.074055
79	Co	-0.102976	1.033476
80	Co	-0.030121	0.071904

Sum of atomic charges = 2.000000
Sum of spin charges = 2.000000

Coordinates and energy for singlet [4OAc-4py]$^{2+}$. Energy shown below is for ECPs with wB97M-V.

C	-1.26441	-1.63420	2.80333
C	-1.73646	-2.26334	4.08296
H	-1.72819	-1.49770	4.86591
H	-1.04008	-3.04607	4.37179
H	-2.75291	-2.65365	4.01202
C	1.28674	1.59064	-2.76903
C	1.78688	2.24404	-4.02501
H	2.43750	1.54797	-4.55038
H	2.29534	3.19062	-3.83666
H	0.92325	2.43930	-4.66915
C	-2.14951	2.64217	0.57667
C	-3.06313	3.80721	0.84721
H	-2.89235	4.15575	1.87073
H	-4.10767	3.50896	0.76591
H	-2.83803	4.63218	0.17157
C	2.11811	-2.71155	-0.58998
C	3.07043	-3.84309	-0.84812
H	4.08328	-3.42609	-0.91541
H	2.84052	-4.32981	-1.79784
H	3.04710	-4.55313	-0.01846

C	1.86029	0.43262	3.41412
H	0.79523	0.60295	3.52426
C	2.80639	0.92156	4.29797
H	2.48221	1.48923	5.15925
C	4.15341	0.66630	4.05358
H	4.91509	1.01514	4.74298
C	4.51388	-0.04921	2.91458
H	5.54819	-0.26312	2.67990
C	3.51941	-0.50001	2.06350
H	3.72504	-1.05261	1.15484
C	1.24451	3.45180	1.95488
H	0.17935	3.46209	2.15152
C	2.12705	4.23344	2.68569
H	1.75077	4.86282	3.48391
C	3.48239	4.19392	2.37036
H	4.19596	4.79265	2.92814
C	3.90530	3.37613	1.32588
H	4.94874	3.32549	1.03996
C	2.96487	2.62416	0.63680
H	3.23899	1.98094	-0.19271
C	-3.52748	-2.14541	-0.32772
H	-2.90065	-2.72416	0.34123
C	-4.65529	-2.68657	-0.92766
H	-4.92185	-3.71897	-0.73764
C	-5.43387	-1.87906	-1.75246
H	-6.32431	-2.27434	-2.23158
C	-5.05485	-0.55317	-1.94743
H	-5.63762	0.11464	-2.57179
C	-3.91477	-0.08125	-1.31390
H	-3.59196	0.94851	-1.41661
C	-0.91765	-2.89643	-2.76185
H	-0.36527	-3.43227	-1.99826
C	-1.68939	-3.52694	-3.72354
H	-1.74221	-4.60756	-3.73605
C	-2.36306	-2.75018	-4.66315
H	-2.94880	-3.22473	-5.44299
C	-2.26784	-1.36245	-4.59426
H	-2.78764	-0.72128	-5.29262
C	-1.47465	-0.79445	-3.61305
H	-1.35323	0.27523	-3.48751
N	2.22516	-0.26885	2.33144
N	1.65935	2.65885	0.95028
N	-3.16566	-0.86618	-0.51904
N	-0.80722	-1.55945	-2.73736
O	-0.44877	-1.51584	-0.02504
O	-0.18081	0.59089	1.38061
O	1.51129	0.09361	-0.26269
O	-0.84554	0.60590	-1.10815
O	0.00919	-1.71314	2.59422
O	-2.10907	-1.05841	2.06255
O	-2.62416	1.47533	0.77286
O	-0.96495	2.92230	0.19057
O	1.07565	2.30819	-1.75245
O	1.07551	0.31866	-2.86560
O	1.56032	-2.17894	-1.60667
O	1.96192	-2.35862	0.62800
Co	0.84335	-0.88087	1.13053

Co	-1.63940	-0.12965	0.43652
Co	0.39227	1.60776	-0.09316
Co	0.34596	-0.69879	-1.45647

Final energy: -2790.0401144486

Spin densities for singlet $[4OAc\text{-}4py]^{2+}$

Ground-State Mulliken Net Atomic Charges

Atom		Charge (a.u.)	Spin (a.u.)
1	C	0.105934	-0.003195
2	C	-0.404185	0.004585
3	H	0.212779	-0.000348
4	H	0.207363	-0.000027
5	H	0.205060	0.000500
6	C	0.058935	0.002907
7	C	-0.420296	-0.004455
8	H	0.208531	0.000050
9	H	0.205170	-0.000563
10	H	0.209174	0.000273
11	C	0.253104	-0.000941
12	C	-0.385827	0.000153
13	H	0.205480	0.000060
14	H	0.198061	-0.000051
15	H	0.207294	-0.000007
16	C	0.224793	0.000295
17	C	-0.336905	-0.000641
18	H	0.213054	-0.000298
19	H	0.203425	0.000285
20	H	0.213277	0.000169
21	C	0.010165	-0.002092
22	H	0.209631	0.000935
23	C	0.084337	0.003720
24	H	0.189516	0.000141
25	C	-0.226773	-0.004376
26	H	0.199912	0.000223
27	C	-0.072328	0.003008
28	H	0.195339	0.000218
29	C	0.004449	-0.004125
30	H	0.188692	0.000971
31	C	0.021064	-0.000103
32	H	0.184910	0.000066
33	C	-0.128739	-0.002132
34	H	0.192947	0.000028
35	C	-0.242873	-0.000292
36	H	0.197003	0.000015
37	C	0.093188	-0.000243
38	H	0.187252	0.000018
39	C	-0.061147	-0.000963
40	H	0.190483	0.000002
41	C	-0.014107	0.000687
42	H	0.184435	-0.000036
43	C	0.013153	0.001233
44	H	0.190445	-0.000018

45	C	-0.225300	0.000195
46	H	0.197645	-0.000013
47	C	-0.030221	0.001731
48	H	0.189966	-0.000022
49	C	-0.003250	-0.000463
50	H	0.184876	-0.000025
51	C	0.077281	0.003957
52	H	0.191122	-0.001014
53	C	-0.051475	-0.003207
54	H	0.194866	-0.000208
55	C	-0.206593	0.005003
56	H	0.201394	-0.000235
57	C	0.050378	-0.004023
58	H	0.190915	-0.000159
59	C	0.010497	0.003021
60	H	0.201887	-0.001010
61	N	-0.414276	0.033160
62	N	-0.353969	0.004165
63	N	-0.340864	-0.002964
64	N	-0.416222	-0.034099
65	O	0.900596	-0.007328
66	O	0.939764	0.088594
67	O	0.942651	0.006889
68	O	0.949977	-0.079644
69	O	-0.456347	0.005474
70	O	-0.391414	-0.006369
71	O	-0.436252	0.001436
72	O	-0.406602	-0.001373
73	O	-0.425019	0.005198
74	O	-0.436182	-0.008945
75	O	-0.451608	0.001039
76	O	-0.450016	-0.000509
77	Co	-0.453311	-1.211079
78	Co	-0.217196	0.007697
79	Co	-0.161786	-0.004632
80	Co	-0.471088	1.204126

Single crystal x-ray diffraction.

X-ray diffraction data were collected using Bruker AXS diffractometers. Crystals of complexes were mounted on a Kappa geometry goniostat coupled to an APEX-II CCD detector with Mo Kα (λ = 0.71073 Å) generated by a microfocus sealed tube and monochromated by QUAZAR multilayer mirrors. Crystals were kept at 100(2) K throughout collection. Collection strategy determination, integration, scaling, and space group determination were performed with Bruker APEX (v.2, v.3) software, and solutions were obtained with SIR-92 and SHELXT-2014.[7,8] All structures were refined with SHELXL-2014.[9] Molecular structure figures were visualized with ORTEP 3.2. Additional details can be found in the crystallographic information files.

References.

(1) Chakrabarty, R.; Bora, S. J.; Das, B. K. *Inorg. Chem.* **2007**, *46* (22), 9450–9462.

(2) Berardi, S.; La Ganga, G.; Natali, M.; Bazzan, I.; Puntoriero, F.; Sartorel, A.; Scandola, F.; Campagna, S.; Bonchio, M. *J. Am. Chem. Soc.* **2012**, *134* (27), 11104–11107.

(3) Shao, Y.; Gan, Z.; Epifanovsky, E.; Gilbert, A. T. B.; Wormit, M.; Kussmann, J.; Lange, A. W.; Behn, A.; Deng, J.; Feng, X.; Ghosh, D.; Goldey, M.; Horn, P. R.; Jacobson, L. D.; Kaliman, I.; Khaliullin, R. Z.; Kuś, T.; Landau, A.; Liu, J.; Proynov, E. I.; Rhee, Y. M.; Richard, R. M.; Rohrdanz, M. A.; Steele, R. P.; Sundstrom, E. J.; III, H. L. W.; Zimmerman, P. M.; Zuev, D.; Albrecht, B.; Alguire, E.; Austin, B.; Beran, G. J. O.; Bernard, Y. A.; Berquist, E.; Brandhorst, K.; Bravaya, K. B.; Brown, S. T.; Casanova, D.; Chang, C.-M.; Chen, Y.; Chien, S. H.; Closser, K. D.; Crittenden, D. L.; Diedenhofen, M.; Jr, R. A. D.; Do, H.; Dutoi, A. D.; Edgar, R. G.; Fatehi, S.; Fusti-Molnar, L.; Ghysels, A.; Golubeva-Zadorozhnaya, A.; Gomes, J.; Hanson-Heine, M. W. D.; Harbach, P. H. P.; Hauser, A. W.; Hohenstein, E. G.; Holden, Z. C.; Jagau, T.-C.; Ji, H.; Kaduk, B.; Khistyaev, K.; Kim, J.; Kim, J.; King, R. A.; Klunzinger, P.; Kosenkov, D.; Kowalczyk, T.; Krauter, C. M.; Lao, K. U.; Laurent, A. D.; Lawler, K. V.; Levchenko, S. V.; Lin, C. Y.; Liu, F.; Livshits, E.; Lochan, R. C.; Luenser, A.; Manohar, P.; Manzer, S. F.; Mao, S.-P.; Mardirossian, N.; Marenich, A. V.; Maurer, S. A.; Mayhall, N. J.; Neuscamman, E.; Oana, C. M.; Olivares-Amaya, R.; O'Neill, D. P.; Parkhill, J. A.; Perrine, T. M.; Peverati, R.; Prociuk, A.; Rehn, D. R.; Rosta, E.; Russ, N. J.; Sharada, S. M.; Sharma, S.; Small, D. W.; Sodt, A.; Stein, T.; Stück, D.; Su, Y.-C.; Thom, A. J. W.; Tsuchimochi, T.; Vanovschi, V.; Vogt, L.; Vydrov, O.; Wang, T.; Watson, M. A.; Wenzel, J.; White, A.; Williams, C. F.; Yang, J.; Yeganeh, S.; Yost, S. R.; You, Z.-Q.; Zhang, I. Y.; Zhang, X.; Zhao, Y.; Brooks, B. R.; Chan, G. K. L.; Chipman, D. M.; Cramer, C. J.; III, W. A. G.; Gordon, M. S.; Hehre, W. J.; Klamt, A.; III, H. F. S.; Schmidt, M. W.; Sherrill, C. D.; Truhlar, D. G.; Warshel, A.; Xu, X.; Aspuru-Guzik, A.; Baer, R.; Bell, A. T.; Besley, N. A.; Chai, J.-D.; Dreuw, A.; Dunietz, B. D.; Furlani, T. R.; Gwaltney, S. R.; Hsu, C.-P.; Jung, Y.; Kong, J.; Lambrecht, D. S.; Liang, W.; Ochsenfeld, C.; Rassolov, V. A.; Slipchenko, L. V.; Subotnik, J. E.; Voorhis, T. V.; Herbert, J. M.; Krylov, A. I.; Gill, P. M. W.; Head-Gordon, M. *Mol. Phys.* **2015**, *113* (2), 184–215.

(4) Khaliullin, R. Z.; Bell, A. T.; Head-Gordon, M. *J. Chem. Phys.* **2008**, *128* (18), 184112.

(5) Mardirossian, N.; Head-Gordon, M. *J. Chem. Phys.* **2016**, *144* (21), 214110.

(6) Andrae, D.; Häußermann, U.; Dolg, M.; Stoll, H.; Preuß, H. *Theor. Chim. Acta 77* (2), 123–141.

(7) Altomare, A.; Cascarano, G.; Giacovazzo, C.; Guagliardi, A.; Burla, M. C.; Polidori, G.; Camalli, M. *J. Appl. Crystallogr.* **1994**, *27* (3), 435–435.

(8) Sheldrick, G. M. *Acta Crystallogr. Sect. A* **2015**, *71* (1), 3–8.

(9) Sheldrick, G. M. *Acta Crystallogr. Sect. C Struct. Chem.* **2015**, *71* (1), 3–8.
